# DREADD Modulation of Transplanted DA Neurons Reveals a Novel Parkinsonian Dyskinesia Mechanism Mediated by the Serotonin 5-HT6 Receptor

**DOI:** 10.1016/j.neuron.2016.04.017

**Published:** 2016-06-01

**Authors:** Patrick Aldrin-Kirk, Andreas Heuer, Gang Wang, Bengt Mattsson, Martin Lundblad, Malin Parmar, Tomas Björklund

**Affiliations:** 1 Molecular Neuromodulation, Department of Experimental Medical Science, Lund University, 221 84 Lund, Sweden; 2 Wallenberg Neuroscience Center, Lund University, 221 84 Lund, Sweden; 3 Developmental and Regenerative Neurobiology, Department of Experimental Medical Science, Lund University, 221 84 Lund, Sweden

## Abstract

Transplantation of DA neurons is actively pursued as a restorative therapy in Parkinson’s disease (PD). Pioneering clinical trials using transplants of fetal DA neuroblasts have given promising results, although a number of patients have developed graft-induced dyskinesias (GIDs), and the mechanism underlying this troublesome side effect is still unknown. Here we have used a new model where the activity of the transplanted DA neurons can be selectively modulated using a bimodal chemogenetic (DREADD) approach, allowing either enhancement or reduction of the therapeutic effect. We show that exclusive activation of a cAMP-linked (Gs-coupled) DREADD or serotonin 5-HT6 receptor, located on the grafted DA neurons, is sufficient to induce GIDs. These findings establish a mechanistic link between the 5-HT6 receptor, intracellular cAMP, and GIDs in transplanted PD patients. This effect is thought to be mediated through counteraction of the D2 autoreceptor feedback inhibition, resulting in a dysplastic DA release from the transplant.

## Highlights

• Bimodal DREADDs and transgenic rats enable remote control of transplant function
• Gs-coupled increase of cAMP using DREADDs leads to graft-induced dyskinesias (GIDs)
• Serotonin 5-HT6 receptor stimulation gives GIDs through dysplastic DA release
• DA neurons grafted to the human brain have high levels of the 5-HT6 receptor

## Teaser

Brain repair through transplantation shows great potential for the future treatment of Parkinson’s disease. Aldrin-Kirk et al. combine fetal transplants from TH-Cre driver rats with bimodal chemogenetic receptors to elucidate a novel mechanism for graft-induced dyskinesias.

## Introduction

Restorative therapies based on nerve cell replacement, from extrinsic or intrinsic sources, have seen significant advances over the past decade. Interest in the therapeutic potential of cell replacement in Parkinson’s disease (PD) in particular has re-emerged with the new EU-funded clinical trial using fetal cells (Abbott, 2014), the development of optimized and efficient differentiation protocols for human embryonic stem cells (hESCs) (Kirkeby et al., 2012, Kriks et al., 2011), and the emerging plans for the use of hESCs or induced pluripotent stem cells (iPSCs) in clinical trials in PD patients (http://www.gforce-pd.com/). Pioneering clinical trials, performed over the last decades using dopamine (DA) neuroblasts from fetal mesencephalon (still considered the gold-standard DA cell replacement in PD) have been encouraging but also raised significant concerns. While some grafted patients have displayed substantial long-term clinical benefit from the dopaminergic cell transplants placed in the caudate/putamen (Kefalopoulou et al., 2014), the outcome has been highly variable (Barker et al., 2013), and a significant number of patients have also developed abnormal involuntary movements induced by the graft (graft-induced dyskinesias, or GID). This troublesome side effect, seen in the absence of any drug treatment, has so far not been possible to reproduce in rodent or primate models of PD.

Recent studies have shown that these GIDs are dependent of serotonergic neurotransmission (Politis et al., 2011) and that they can be suppressed by drugs acting on inhibitory autoreceptors located on the serotonin neurons (Politis et al., 2010). However, the functional link between the serotonin system and dysregulated DA neurotransmission causing dyskinesias still remains elusive. The main reason for this is due to the fact that all attempts to reproduce this side effect in an authentic and clinically relevant animal model have so far failed.

The goal of this study was to explore the mechanism underlying the induction of GIDs using a novel rat model where DA neurons from Cre-expressing donor rats (a knockin Cre driver line under the endogenous TH [tyrosine hydroxylase] promoter) are transplanted to the striatum in parkinsonian rats. The transplants are subsequently transduced to express, selectively within the grafted DA neurons, a novel bimodal pair of chemogenetic receptors (designer receptors exclusively activated by designer drugs, or DREADDs) (Vardy et al., 2015). While these DREADDs have been shown to either increase or silence (depending on the DREADD expressed) axonal firing in DA neurons in vivo and in slice preparations (Mahler et al., 2014, Marchant et al., 2016, Wang et al., 2013), this model enables us for the first time to selectively regulate the activity of grafted DA neurons. This has allowed us to unequivocally determine the functional impact of ectopically transplanted DA neurons (placed in the striatum, which is the target structure for dopaminergic transmission from the A9 midbrain DA neurons and corresponds to the placement in caudate/putamen in patients). While previous studies have managed to silence a tonically active hESC transplant using optogenetic tools in mice (Steinbeck et al., 2015) or to activate reprogrammed fibroblasts (mouse-derived induced neurons) using DREADDs in rats (Dell’Anno et al., 2014), this study aims to achieve a selective and reversible bimodal regulation of the DA neurons contained in the transplant, imperative to dissect the underlying mechanisms of the GIDs.

In this study, we show that in vivo transduction of transplanted Cre-expressing neurons using recombinant adeno-associated viral (AAV) vectors carrying a Cre-inducible construct can be achieved with very high efficiency and that this method can be used to selectively regulate DA neuron function in a bimodal manner. With this model, we demonstrate unequivocally that an active DA neurotransmission is necessary for the therapeutic capacity of the fetal graft. Our data also point to the fact that the ectopic placement of the graft may result in a suboptimal function, and that this can be significantly enhanced using selective activation of the DA cells in the graft.

We found that increase of cyclic AMP (cAMP) in the grafted DA neurons, obtained through activation of the metabotropic Gs-coupled DREADD (rM3Ds), is sufficient to induce significant GIDs in animals in the absence of any L-DOPA treatment. Building on these findings, we were able to identify the serotonin 5-HT6 receptor as an important activator of DA neurons in the graft and show that selective activation of this receptor using the potent 5-HT6 agonist ST1936 can induce significant GIDs with a phenotype very similar to L-DOPA-induced dyskinesias (LIDs) even in unprimed animals (i.e., animals that never received L-DOPA and thus never developed LIDs). Lastly, we show that this receptor is highly relevant for clinical transplantation as postmortem analysis of human fetal cell transplants to the putamen of PD patients shows expression of the receptor, as do human DA neurons grafts obtained from human fetal brain or differentiated hESCs.

## Results

### Fetal Transplants from Knockin Cre Driver Rats Reinnervate the Parkinsonian Striatum and Result in Time-Dependent, Partial Recovery of Motor Function

**Figure 1:**
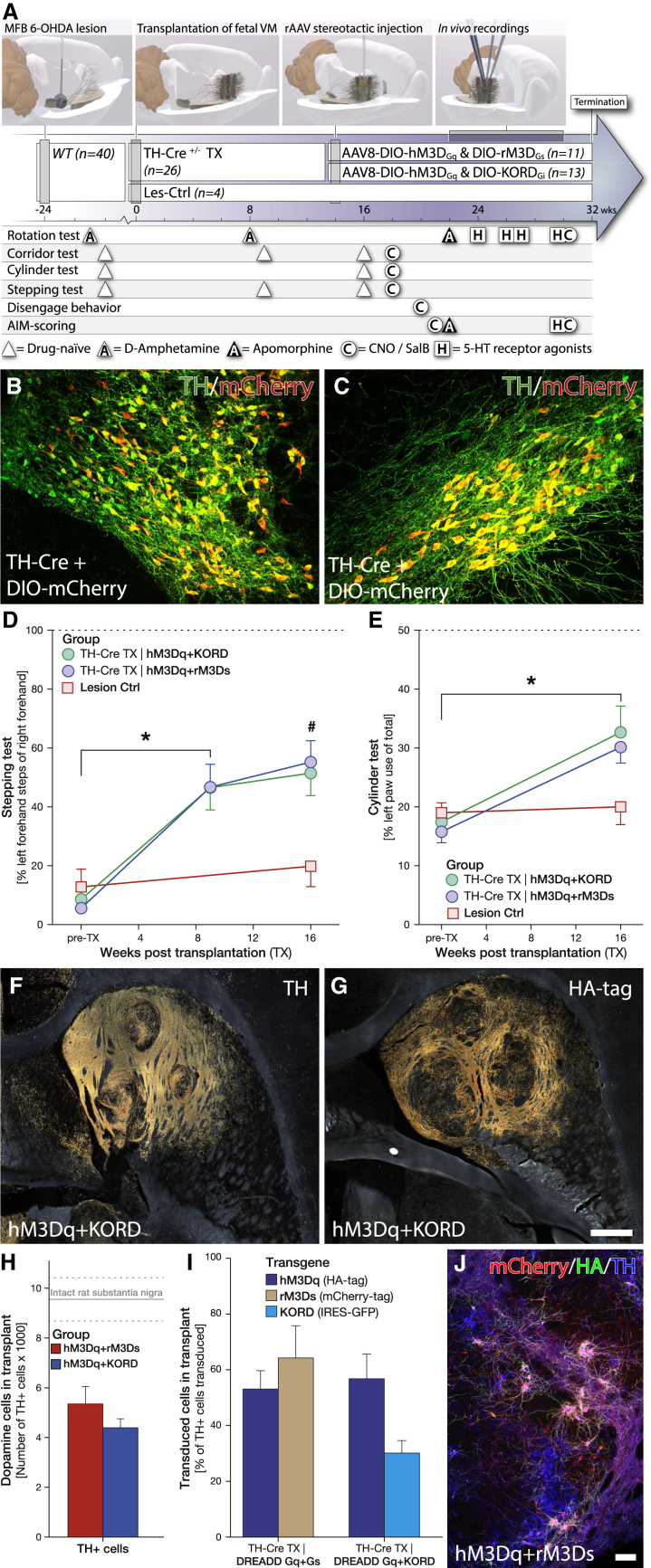
Characterization of Dopaminergic Fetal Grafts and AAV-Mediated Transduction Efficacy (A) Experimental timeline illustrating the time points for all major surgical procedures and behavioral tests for animals expressing hM3Dq/rM3Ds and hM3Dq/KORD vectors. (B and C) LSM of midbrain dopaminergic neurons, virally transduced with AAV-8 DIO-mCherry and stained for TH (green) and mCherry (red). VTA (B) and SN (C) dopaminergic neurons displayed high specificity for mCherry-labeled neurons, demonstrating that the TH-Cre knockin rat has highly selective expression of Cre recombinase, localized to dopaminergic neurons. (D and E) Motor recovery following grafting of dopaminergic fetal cells in the stepping (D) and cylinder (E) behavior tests. Dashed line in graphs represents the average performance of an unlesioned rat (see also Figure S1). Lesioned animals receiving the fetal midbrain progenitor transplants (TX) recovered significantly, yet not completely, in these tests of advanced motor function, while there was no spontaneous motor recovery observed in lesioned rats that were not transplanted (Lesion Ctrl). (F and G) Dark field microscopy imaging of three-site dopaminergic grafts in horizontal sections, using DAB-amplified immunohistochemical detection of the DA marker TH (F) and characterization of AAV transduction efficacy using HA-tag antibody (detecting the tagged hM3Dq transgene) (G) (see also Figure S2). (H) Stereological quantification of dopaminergic neurons within the grafts of both hM3Dq + rM3Ds (red) and hM3Dq + KORD (blue) transduced animals in relation to intact midbrain dopaminergic neurons of the SN pars compacta (dashed lines ± 1 SD). (I) Quantification of AAV-transduced grafted neurons for the three vector constructs: hM3Dq-HA-tag (blue), rM3Ds-mCherry (tan), and KORD-GFP (cyan) for both groups of hM3Dq + rM3Ds and hM3Dq + KORD transplants, presented as transduction efficiency related to the number of TH+ transplanted DA neurons. (J) LSM image of grafted neurons stained for rM3Ds-mCherry (red), hM3Dq-HA (green), and TH (blue) (see also Figure S2). Scale bar, 500 μm (G) and 100 μm (J). All values reported as arithmetic mean ± 1 SEM. ^∗^p < 0.05 in Bonferroni corrected paired Student’s t test; #significantly different from control (p < 0.05) in repeated-measures ANOVA, followed by Bonferroni corrected one-way ANOVA, followed by Dunnett’s T3 post hoc test.

The experimental setup of this study (Figure 1A) was designed to allow for both the assessment of unperturbed therapeutic capacity of fetal DA neuroblasts after transplantation (TX) and for the assessment of chemogenetic activation (hM3Dq [Gq coupled] and rM3Ds [Gs coupled]) and silencing (KORD [Gi coupled]). The main activating DREADD combination, rM3Dq/hM3Ds, was selected to enable drive of the DA neurons with a simultaneous counteracting activity on the D2 autoreceptors. The Gi-coupled D2 receptor reduces intracellular cAMP when the synaptic DA concentration increases, with the result that the activation threshold for further release is increased. The activation of the hM3Ds actively counteracts this effect. The novel KORD receptor was matched with the hM3Dq to enable in vivo studies of bimodal regulation where one ligand could induce DA release (using CNO) and the other inhibit DA release (using salvinorin B, or SalB). These two groups, together with the rM3Ds/KORD group presented in Figure 6, enable us to compare the action of Gs- and Gq-coupled G protein activation in DA neurons. The DREADDs were specifically expressed in the mature DA neurons of the transplant through the induction of gene transcription using the Cre-loxP system. The Cre-recombinase was selectively expressed in the DA neurons of the graft through sourcing of TH-Cre ± fetal tissue from wild-type (WT) females bred with male TH-Cre homozygous knockin rats. The specificity of Cre expression was carefully examined in the adult TH-Cre rat to confirm that the Cre expression is restricted to only DA neurons in the ventral midbrain (Figures 1B, 1C, and S1B, available online). Unilateral grafting of fetal dopaminergic neurons into the striatum of 6-OHDA lesioned rats resulted in significant, yet partial, recovery in the stepping and cylinder motor performance tests (Figures 1D and 1E), as well as recovery in sensorimotor neglect and the typical overcompensation in amphetamine-induced rotations (Figures S1C and S1D). Selective expression of transgenes in the grafted DA neurons was efficiently achieved throughout the grafts using Cre-inducible AAV8 vectors injected in the centroid between the 3 × 2 μl transplant deposits (Figures 1F and 1G). Quantification of TH-positive dopaminergic neurons within the three graft tracts confirmed successful integration of a large number of graft-derived DA neurons into the host tissue, representing approximately 50% of the uninjured rat substantia nigra (SN) (Figures 1F and 1H). Graft-derived, dopaminergic projections (assessed specifically using the Cre-activated HA-tagged protein) were mainly localized to the striatum but were also found in the cerebral cortex and in the reverse direction along the medial forebrain bundle through the globus pallidus, with projections reaching as far as the SN (Figures 1G and S2A–S2E).

### Fetal Transplants from Knockin Cre Driver Rats Allow for Cell-Specific Targeting In Situ Using DIO-AAV8 Vectors

**Figure 2:**
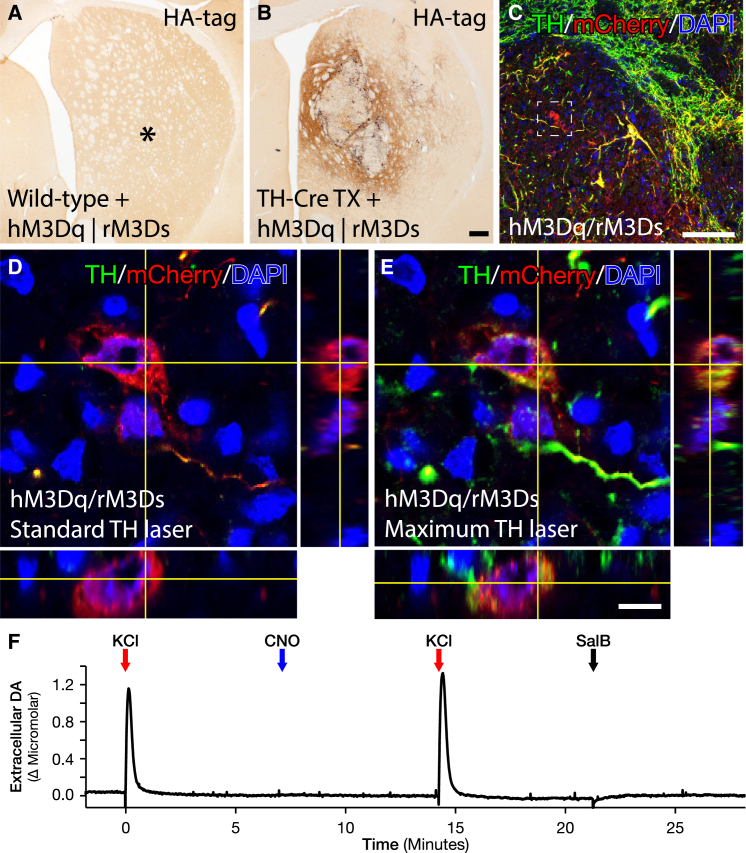
In Vivo Specificity Evaluation of DIO-AAV Vectors to Transplanted TH-Cre-Positive Neuroblasts and Off-Target In Vivo Activation of the DREADD Ligands (A and B) Expression of DIO-hM3Dq and DIO-rM3Ds DREADDs, following viral infusion into the striatum of WT animals (A) and animals grafted with dopaminergic fetal tissue from TH-Cre transgenic rats (B). ^∗^Denotes position of viral infusion (see also Figure S3). (C–E) Assessment of virally transduced, grafted mCherry-positive neurons that appeared TH negative under normal laser power conditions. (C) Overview of dopaminergic fetal graft within the striatum with an mCherry-positive neuron that appeared TH negative (white box). When comparing high-magnification z stacks of these mCherry-positive grafted neurons under normal laser power conditions (D) with high laser power conditions (E), it is clear that these mCherry-positive (red) neurons are indeed TH positive (green), although at low levels. We hypothesize based on cell morphology and heterogeneous TH expression that these are grafted neurons originating from the VTA (see also Figure S1). (F) Control measurements in the intact striatum using electrochemical chronoamperometric recordings of DA release. Local KCl administration (red arrow) evoked strong DA release while neither CNO (blue arrow) nor SalB (black arrow) administration resulted in any measurable increase in extracellular DA. Scale bar, 200 μm (B), 100 μm (C), and 10 μm (E).

Chemogenetic modulation of neuronal activity of the graft-derived TH neurons was achieved using three AAV8 vectors delivering the activation and inhibiting DREADD constructs in Cre-inducible (DIO, or double-floxed inverted orientation) expression cassettes. Histological analysis revealed that all three graft deposits could be transduced with a single stereotactic injection, achieving robust and highly specific transduction of TH-positive neurons of all three transgenes (Figures 1G, 1I, 1J, and S1E–S1L). The specificity and off-target function of this vector were evaluated in vivo in the striatum of WT rats without Cre expression (Figures 2A, 2B, S3A, and S3B) using both behavior test and postmortem analyses to confirm that both the vectors and the utilized ligands are biologically inert in the absence of Cre (Figures 6B and 6C). Of note is that the expression strength of the AAV-DIO-derived transgene does not correlate with TH (and thereby Cre) expression as it is driven by the pan-neuronal promoter Synapsin-1 and is strongly expressed also in DA neurons with TH levels very near the detection limit (Figures 2C–2E and S3C–S3H; Stuber et al., 2015). The following AAV8 DREADD vectors were combined to allow for two types of bimodal regulation: Gq- and Gs-coupled activation using the same activating ligand (CNO, hM3Dq + rM3Ds, n = 11), or Gq-coupled activation using the CNO ligand and Gi-coupled inhibition using the novel orthogonal ligand SalB (hM3Dq + KORD, n = 13). In addition, the Gs was evaluated without the Gq (rM3Ds + KORD, n = 6), and a novel rat-codon-optimized, M4-based Gi DREADD was developed (increasing sequence homology from 90% to 98.2% without changes in the amino acid sequence) and utilized to evaluate the inhibitory function of the Gi on the function of the graft (rM4Di + eYFP, n = 6) (Figure 6). Both ligands CNO and SalB were confirmed to be otherwise biologically inert at the doses administered (Figures 2F, 6B, 6C, and S6).

### Chemogenetic Modulation of DA Grafts Using First and Second Generation DREADDs Enables Functional Potentiation and Silencing in the Same Animal

**Figure 3:**
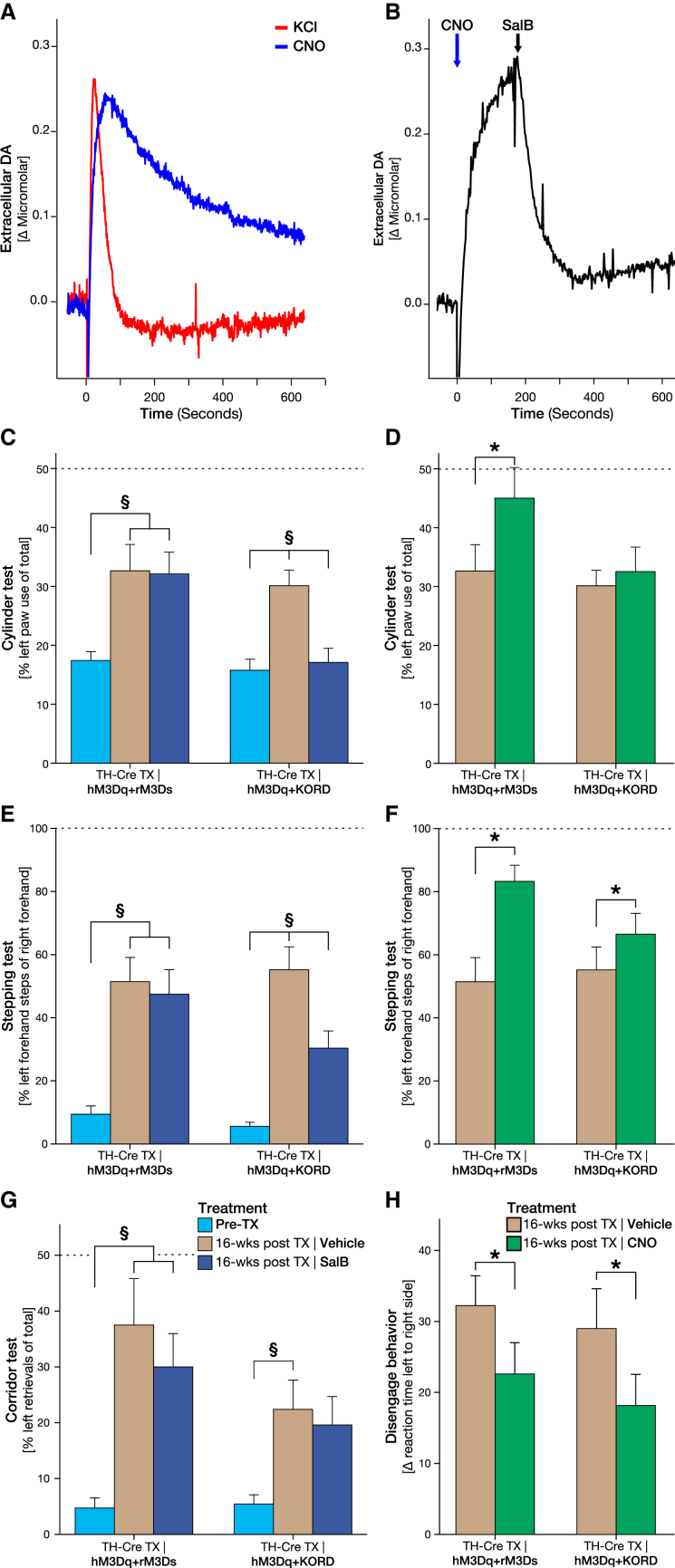
Bidirectional Modulation of DA Release from Grafted Neurons Using DREADDs (A) Chronoamperometric in vivo recordings in the striatum (in proximity to the transplantation sites), comparing DA release following local administration of KCl (red) and CNO (blue) from the dopaminergic graft. The average peak DA concentration after CNO (1.08 ± 0.51 μM) was comparable to the average KCl-induced DA release (1.60 ± 0.41 μM). (B) In vivo recordings of bidirectional DA modulation from hM3Dq + KORD-expressing graft where CNO (blue arrow)-induced DA release could be silenced following administration of SalB at assumed peak of release (black arrow). The average peak DA concentration on CNO was 1.49 ± 0.20 μM compared to 0.67 ± 0.11 μM when combined with inhibition of the graft using SalB. (C and D) Systemic, noninvasive modulation of the dopaminergic graft during assessment in the cylinder task comparing unmodulated graft baseline (tan) to lesion baseline (cyan) and 15 min following SalB treatment (blue). Inhibition of function occurred in hM3Dq + KORD animals, but not in animals lacking the KORD receptor (C), while CNO treatment (green) significantly increased motor performance in hM3Dq + rM3Ds animals (D). (E and F) Bidirectional modulation of motor behavior in the stepping task with hM3Dq + KORD animals reducing motor recovery following SalB treatment (E) and increasing motor recovery following CNO treatment (F), while the ameliorating effect of the hM3Dq + rM3Ds transplants could be increased by treatment with CNO (F) and remained unaltered after SalB administration (E). (G and H) Sensorimotor performance in response to treatment with SalB and CNO in the corridor task (G), and therapeutic potentiation after CNO in a complex sensorimotor integrative task, measured as the decrease in response latency in the disengage test (H) (see also Figure S3). All values reported as arithmetic mean ± 1 SEM. ^∗^p < 0.05 in Bonferroni corrected paired Student’s t test; §p < 0.05 in two-way mixed-model ANOVA followed by Tukey’s post hoc.

Local administration of CNO was able to produce a specific and repeatable DA release from the transplants of a peak amplitude similar to that of KCl (as measured by in vivo electrochemical detection methods) in animals receiving both vector mixes, but with slower reuptake rate (Figures 3A, 5D, S6B, and S6F). In animals receiving the hM3Dq + KORD combination, local administration of SalB 60 s before KCl injection could strongly suppress the KCl-induced DA release (Figures S6D and S6H). The inhibition was also rapid enough to preemptively terminate the Gq-mediated release, demonstrating in vivo bidirectional modulation (Figure 3B). Of note is that the recording of the DA release was performed at the centroid between the transplant deposits very close to the site of AAV injection to ensure that the release from terminals is recorded and that the highest fraction of transplanted DA neurons is transduced at the site of recording; i.e., a higher transduction rate is expected at the site of recording than that presented in Figure 1I.

After systemic CNO administration, animals expressing the hM3Dq + rM3Ds combination displayed significantly improved motor performance in both the cylinder task and the stepping test in response to CNO, with some animals returning to prelesion levels (Figures 3D and 3F). In contrast, graft-induced recovery in these tests was abolished in animals with transduced hM3Dq + KORD grafts, following treatment with SalB, suppressing the therapeutic effect of the transplant entirely in the cylinder test and to a significant portion in the stepping test (Figures 3C and 3E), but not in the corridor test (Figures 3G and S4A). Interestingly, CNO treatment was sufficient to increase the performance in a complex sensorimotor integrative task, the disengage task that is earlier known to be only marginally improved by DA neuron grafts (Mandel et al., 1990, Winkler et al., 2000) (Figures 3H and S4B). The therapeutic effect was measured as decreased response latency and was observed in both hM3Dq + rM3Ds and hM3Dq + KORD animals after CNO administration.

### Increases in cAMP in DA Neurons of the Graft Selectively Modulate Involuntary Movements

**Figure 4:**
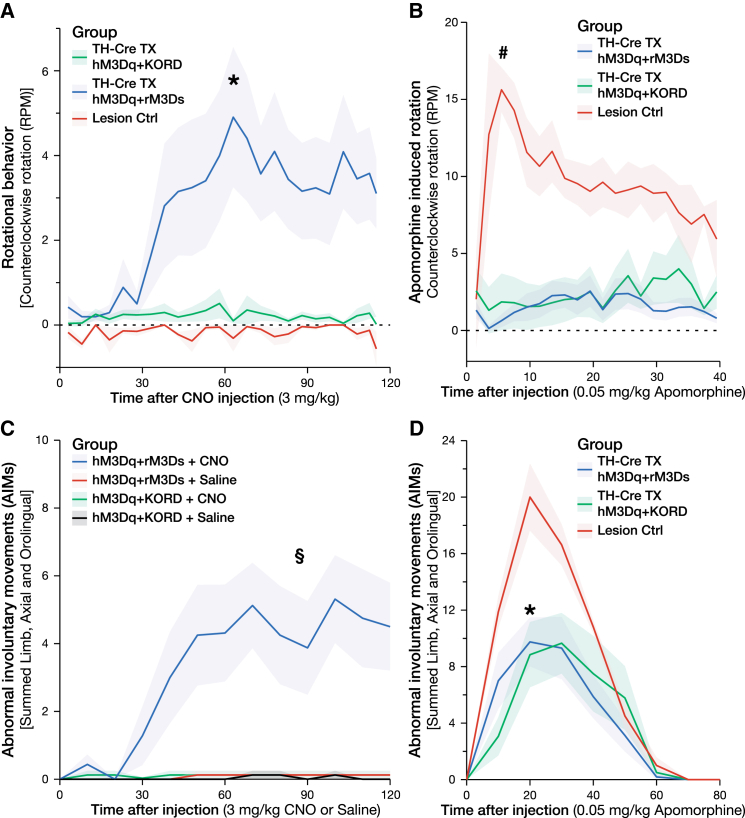
GIDs in Animals with CNO-Activated hM3Dq + rM3Ds Receptors in the DA Transplant (A) Rotational asymmetry was prominent when activating the DA neurons in the transplant of hM3Dq + rM3Ds animals with CNO, but not hM3Dq + KORD or Lesion Ctrls. (B) Rotational behavior induced by apomorphine, however, showed a strong contralateral rotation in Lesion Ctrl animals (red), which was comparatively reduced in grafted hM3Dq + rM3Ds (blue) and hM3Dq + KORD (green) animals, indicating a reduced postsynaptic DA receptor supersensitivity, normalized by the graft. (C) When observed in a home-cage-like environment, treatment with CNO induced AIMs in the hM3Dq + rM3Ds animals only. (D) Apomorphine injections, on the other hand, induced strong AIMs in Lesion Ctrl animals, which was significantly reduced in grafted hM3Dq + rM3Ds and hM3Dq + KORD animals. All values reported as arithmetic mean ± 1 SEM. ^∗^AUC (area under the curve) significantly different from the Lesion Ctrl group, #AUC significantly different from both treatment groups, p < 0.05 using one-way ANOVA followed by Dunnett’s T3 post hoc. §AUC significantly different from all other groups, p < 0.05 using one-way ANOVA followed by Tukey’s post hoc.

**Figure 5:**
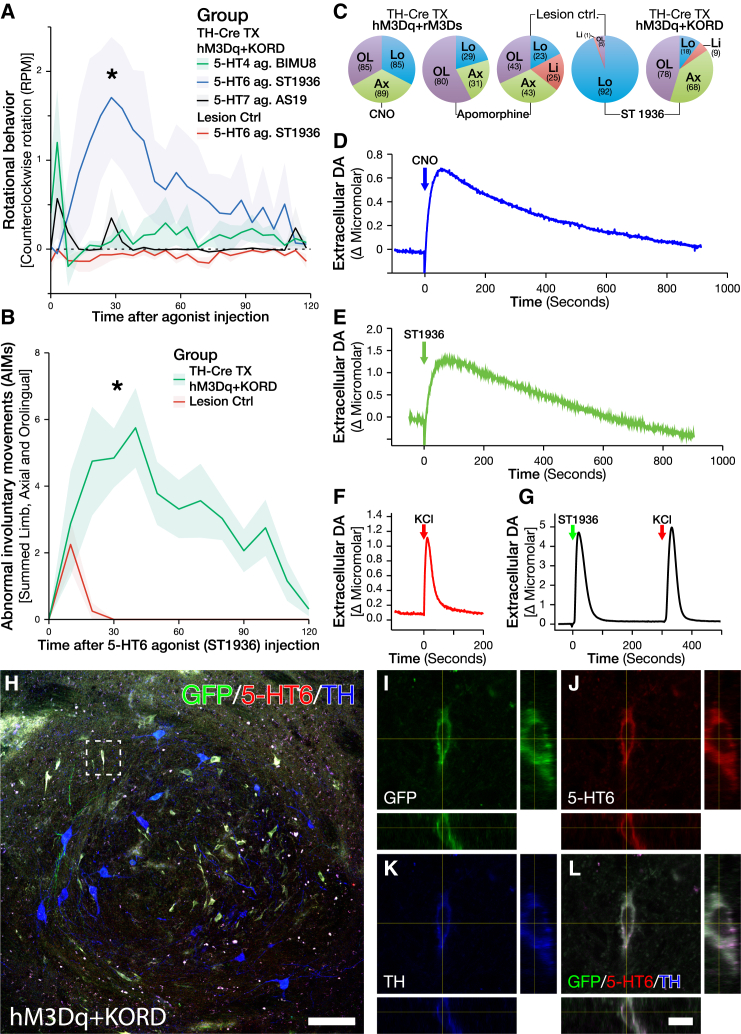
Treatment with 5-HT6 Agonist ST-1936 Induces DA Release and GIDs through Direct Action on the Grafted DA Neurons (A) Rotational behavior following treatment of 5-HT agonists specifically targeting each of the three metabotropic, Gs-coupled, serotonin (5-HT) receptors, with 5-HT4 agonist BIMU8 (green) and 5-HT7 agonist AS19 (black) not inducing any rotational behavior, while 5-HT6 agonist ST-1936 (blue) induced a strong contralateral rotation in grafted hM3Dq + KORD animals (L-DOPA naive and not previously displaying GIDs on CNO), but not Lesion Ctrls (red). (B) Treatment with 5-HT6 agonist ST-1936 induced substantial AIMs in grafted hM3Dq + KORD animals (green) with only a weak, transient AIM score in Lesion Ctrls (red). (C) Comparison of the different manifestations of AIMs between treatments with CNO, apomorphine, and ST-1936. CNO induced mainly locomotor (blue), axial (green), and orolingual (purple) AIMs in hM3Dq + rM3Ds animals. Grafted animals displayed a marked reduction in AIMs following treatment with apomorphine, with limb AIMs (red) being abolished. AIMs following treatment with ST-1936 induced mainly orolingual and axial AIMs, with a relatively small proportion of limb and locomotor AIMs in hM3Dq + KORD-grafted animals. In contrast, Lesion Ctrls mainly exhibited locomotor AIMs with a very small proportion of AIMs being orolingual. (D–G) Electrochemical recordings of DA release in response to local ligand administration, with CNO (D) and ST-1936 (E) inducing a prolonged DA release with very similar kinetics in hM3Dq + KORD-grafted animals, while both KCl (F) and ST-1936 (G) induced a short spike of DA with very different kinetics when applied in the intact, nongrafted striatum (see also Figure S6). (H) LSM image showing strong expression of 5-HT6 (red) colocalized with virally transduced (green) TH (blue)-positive neurons within the dopaminergic fetal graft. (I–L) Magnified z stack of a dopaminergic neuron (taken from dashed square in H) showing the colocalization between virally transduced KORD expression (I), 5-HT6 (J), and TH (K) together with an overlay composite (L) (see also Figure S5). All values reported as arithmetic mean ± 1 SEM. ^∗^AUC significantly different from the Lesion Ctrl group, p < 0.05 using Kruskal-Wallis test followed by Bonferroni corrected all-pair comparison, using the Mann-Whitney U test in (A) and using Student’s t test in (B). Scale bar, 100 μm (H) and 10 μm (L).

**Figure 6:**
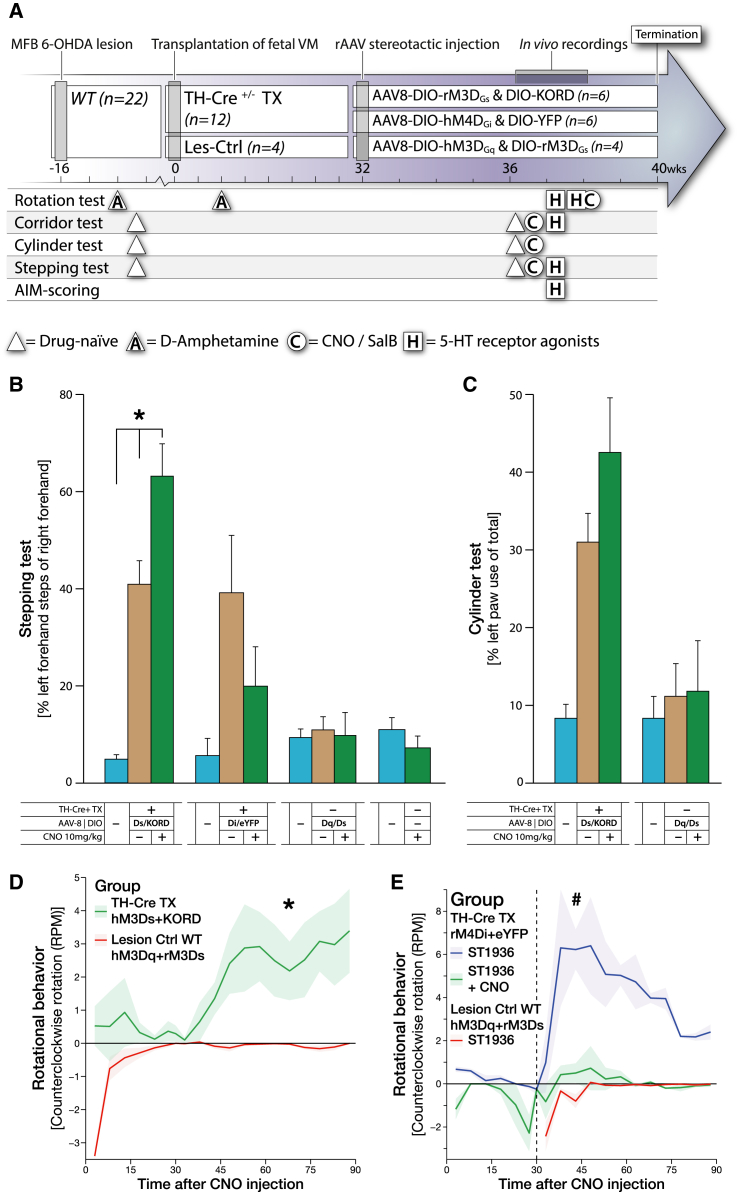
Second Experimental Group: Validation of the rM3Ds DREADD and Reversal of the 5-HT6-Induced Involuntary Movements by rM4Di DREADD (A) Experimental timeline illustrating the time points for all major surgical procedures and behavioral tests for animals expressing rM3Ds/KORD and rM4Di/eYFP vectors. (B and C) Noninvasive modulation of the dopaminergic fetal graft during assessment in the stepping and cylinder tasks, comparing unmodulated lesion baseline (cyan) to graft baseline (tan) and 1 hr following CNO treatment (green). (B) Comparison between grafted rM3Ds/KORD- and rM4Di/eYFP-expressing animals, as well as nongrafted hM3Dq/rM3Ds transduced controls and nongrafted, nontransduced controls. rM3Ds receptor alone significantly potentiates motor performance in response to CNO treatment, while inert in hM3Dq/rM3Ds nongrafted animals. Inhibition of motor function occurred in animals expressing hM4Di in response to CNO in the stepping task. (C) Comparison between grafted rM3Ds/KORD and hM3Dq/rM3Ds transduced nongrafted controls in the cylinder task. (D) Rotational behavior in response to CNO (10 mg/kg) in grafted rM3Gs/KORD animals compared to hM3Dq/rM3Ds transduced nongrafted controls. (E) Rotational behavior in response to 5-HT6 agonist ST-1936 (20 mg/kg) in grafted animals and inhibition of this behavior by the CNO-dependent hM4Di DREADD. CNO treatment effectively abolished ST-1936-mediated rotational behavior, suggesting that 5-HT6-mediated rotational behavior in grafted animals is DA dependent. All values reported as arithmetic mean ± 1 SEM. ^∗^AUC significantly different from the Lesion Ctrl group, p < 0.05 using Student’s t test, #AUC significantly different from both TH-Cre TX rM4Di+eYFP +ST1936 +CNO and Lesion Ctrl WT hM3Dq+rM3Ds +ST1936, p < 0.05 using one-way ANOVA followed by Dunnett’s T3 post hoc.

During the acquisition of the behavior modulation data after CNO administration, hM3Dq + rM3Ds animals were observed to exhibit abnormal body posture, rotational locomotion, and spontaneous limb activity at the peak dose of CNO. hM3Dq + rM3Ds, but not hM3Dq + KORD, animals were found to display a significant and dose-dependent rotational bias away from the transplanted hemisphere (contralateral rotation) in response to CNO (Figures 4A and S4C). When observed in an unconstrained home-cage environment, the same treatment group displayed a broader spectrum of abnormal involuntary movements (AIMs), including both axial and orolingual repetitive movements, peaking around 60 min following CNO treatment and lasting for longer than 2 hr (Figures 4C and 5C; Movie S1). Interestingly, neither the hM3Dq + KORD animals nor the lesion control animals developed any rotational behavior or AIMs in response to CNO. Thus, increase in cAMP, the principle intracellular activation mechanism of Gs-coupled G protein receptors, may be a critical mechanism behind GIDs. Indeed, when activating the graft by CNO-induced increase of cAMP using the rM3Ds DREADD alone, the rotational asymmetry was found to the same magnitude (Figure 6D).

To confirm that these findings were indeed due to presynaptic dysregulation and not postsynaptic receptor sensitivity, animals were treated with the DA agonist apomorphine. While the lesion control animals remained highly supersensitized to apomorphine in the ipsilateral striatum, both hM3Dq + rM3Ds and hM3Dq + KORD displayed significantly reduced rotational behavior and AIMs, consistent with graft-mediated reduction of postsynaptic receptor sensitivity (Figures 4B and 4D).

### Selective Activation of the 5-HT6 Receptor Is Sufficient to Induce GIDs

As noted above, both clinical and preclinical evidence point to the serotonergic neurotransmission as an upstream event required for the maintenance of GIDs. However, the mechanism through which this activation is inducing dysplastic events via the graft remains elusive. Three of the seven families of serotonergic (5-HT) receptors present in the CNS are metabotropic and signal through the Gs cascade: 5-HT4, 5-HT6, and 5-HT7. Therefore, selective agonists toward these receptors were evaluated in the hM3Dq + KORD-treated animals, as they had yet to show any rotational asymmetry or AIMs. While the 5-HT4 (BIMU8) and 5-HT7 (AS19) agonist did not induce any rotational asymmetry, the selective 5HT-6 agonist ST-1936 induced both contralateral rotational behavior as well as AIMs very similar to that observed following CNO treatment (Figures 5A and 5B). Electrochemical recordings of DA release in vivo in response to locally administered ST-1936 revealed a strong release of DA from the transplant with release kinetics strikingly similar to those evoked by the local CNO release with an initial fast response, followed by a slow, gradual reduction in DA levels, indicating that DA release evoked by CNO and ST-1936 may be due to a similar mechanism (Figures 5D and 5E). The other 5-HT agonists, BIMU8 and AS19, did not induce DA release upon local administration in the transplant or in the intact striatum at the highest concentrations used (100 μM) (data not shown). Although locally administered ST-1936 to the intact, nongrafted striatum did evoke DA release, the release pattern of DA was drastically different, with a fast spike of DA that quickly returned to baseline levels, very similar to that of local KCl administration (Figures 5F, 5G, and S6J). In order to confirm that the mechanism of action from ST-1936 is mediated through the DA neurons of the transplant, the rotational asymmetry was re-evaluated in a transplant group receiving the de novo-generated inhibitory DREADD rM4Di, which confers an inhibition of neuronal activity much longer than the KORD. In these animals, we found that the ST-1936-induced rotation could be abolished through pretreatment with CNO (Figure 6E). To confirm that the 5-HT6 agonist could act directly on the grafted DA neurons, brain sections from grafted animals were triple-stained for 5-HT6 receptor, TH, and a Cre-induced marked (GFP). A subgroup of TH-positive neurons was observed that stained strongly positive for the 5-HT6 receptor. These cells were mainly of a smaller rounded morphology but were present all through the graft (Figures 5H–5L and S5A–S5D). Midbrain dopaminergic neurons on the nonlesioned side of these animals did also stain positively for the 5HT-6 receptor, although at a much lower level and with more diffuse staining pattern (Figures S5E–S5H).

**Figure 7:**
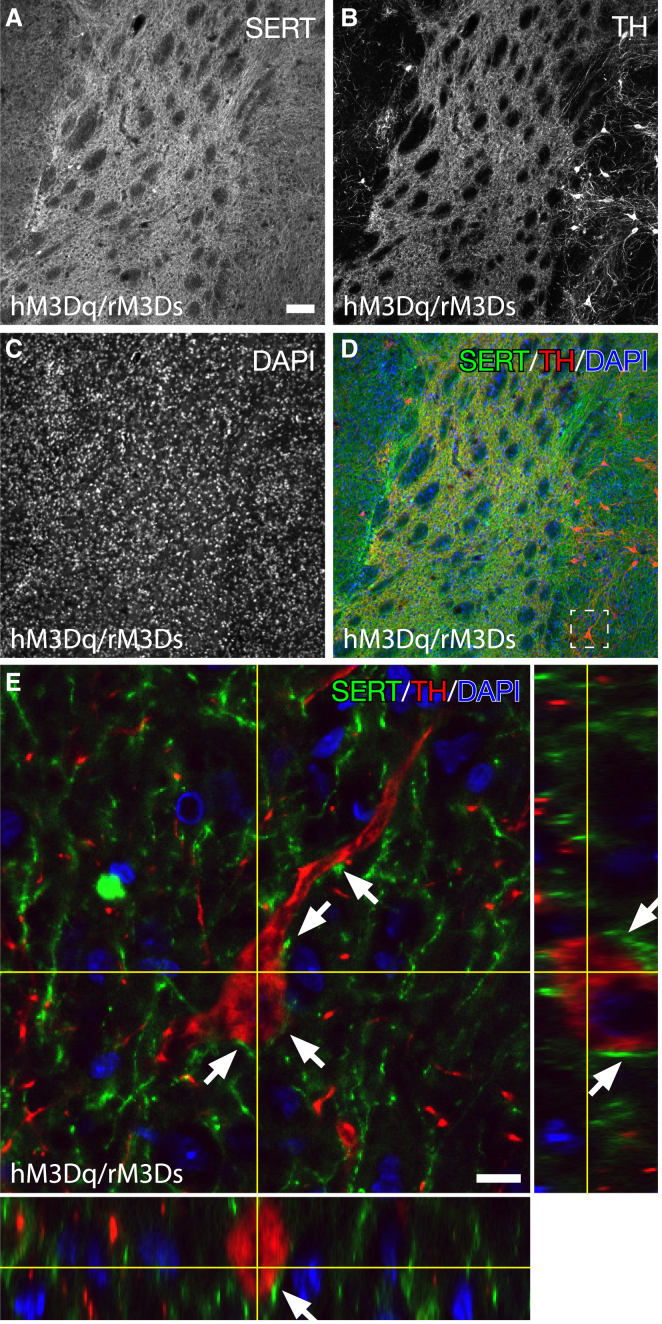
Host- and Graft-Derived Serotonin Neurons Densely Innervate the DA Neurons of the Transplant (A–D) Overview immunofluorescence staining of serotonin expression in the striatum of animals that received dopaminergic fetal grafts, using LSM, stained for (A) SERT, (B) TH, and (C) DAPI with an overlay composite (D). Serotonin projections were as expected—abundant in the striatum with SERT-positive projections infiltrating the grafted tissue. (E) High-magnification z stack of grafted dopaminergic neuron (red) in close proximity to infiltrating SERT-positive (green) projections (white arrows). SERT-positive projections were found to be within close range around grafted dopaminergic neurons, suggesting communication between the two. Scale bars, 100 μm (A) and 10 μm (E).

Previous studies have reported that transplants derived from fetal ventral mesencephalon (VM) can contain variable numbers of 5-HT neurons (Carlsson et al., 2007) and that 5-HT hyperinnervation of the transplanted striatum may contribute to GIDs (Politis et al., 2010). Using high-resolution confocal microscopy, we also found that 5-HT transporter (SERT)-positive neurons indeed exist in the transplant and that both the transplant and the host striatum are densely innervated by 5-HT projections (Figures 7A–7D). At no time was the SERT found in TH-positive cells of the transplant, confirming that these are two distinct populations. Furthermore, the SERT-positive projections densely contacted the transplanted DA neurons with a large number of SERT-positive varicosities touching the DA cell soma, further suggesting that there is a likely environment inside the graft for serotonergic neurotransmission to be a regulator of DA neuron activity acting though the 5-HT6 receptor (Figure 7E).

### The 5-HT6 Receptor Is Also Strongly Expressed in DA-Neuron-Rich Grafts When Transplanted to the Human Brain

**Figure 8:**
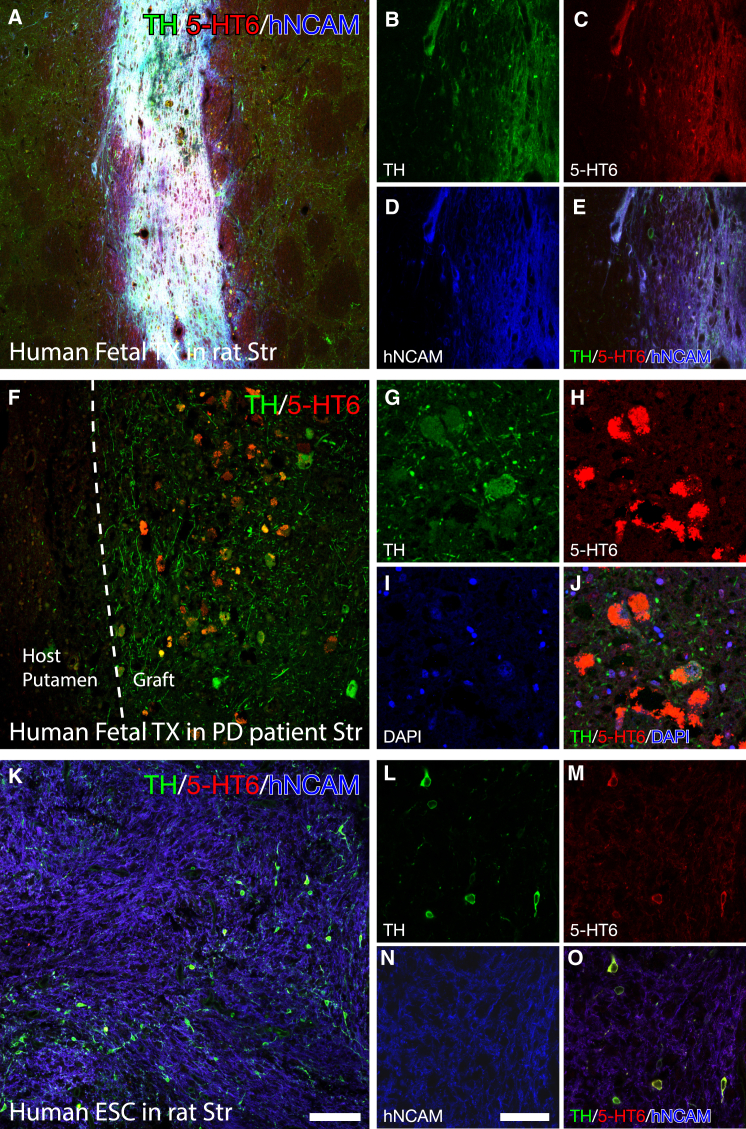
The 5-HT6 Receptor Is Highly Expressed in Human Tissue Grafts Originating from Both Fetal and hESC Sources (A–E) Immunofluorescence staining of 5-HT6 expression in a human dopaminergic fetal graft within the rat striatum (A and C) with the phenotypic staining for TH (A and B), and the identification of human NCAM (A and D), imaged using LSM. The 5-HT6 expression was confirmed to reside in DA neurons originating from the transplanted tissue (E). (F–J) Similarly, immunofluorescence staining of a paraffin-embedded section originating from the putamen of a PD patient that received a dopaminergic fetal graft, imaged using LSM, confirmed that the 5-HT6 receptor (F and H) is highly expressed in the TH-positive (F and G, with overlay in J) DA neurons originating from the fetal graft. Nuclear staining (DAPI) confirmed the 5-HT6 expression to be abundant in the neuronal soma (I). (K–O) To confirm if this receptor is also abundant in DA neurons differentiated from hESCs, a graft derived from the H9 hESC line, differentiated using the novel floor-plate-based protocol and transplanted to the parkinsonian striatum of a nude rat, was evaluated using LSM with immunofluorescence for the same genes: 5-HT6 (K and M), TH (K and L), and the human NCAM (K and N). This confirmed that this receptor is highly expressed in DA neurons also differentiated from this cell source, when transplanted to the striatum. Overlay of all three antigens confirmed the 5-HT6 receptor to reside in hESC-derived DA neurons (O). Scale bar, 100 μm (K) and 50 μm (N).

Very little data exist on the expression of the 5-HT6 receptor in DA neurons, especially of human origin. This is of paramount importance, as to date, spontaneous GIDs have only been observed after transplantation of human fetal VM tissue to the human brain. In this experiment, we first utilized human fetal tissue, obtained from 5- to 8-week-old elective terminations of pregnancies wherefrom the dissociated VM was transplanted to the DA-depleted striatum of immunosuppressed rats. When sacrificed 8 weeks post-transplantation, the TH+ cells of this transplant displayed a very strong expression of the 5-HT6 receptor (Figures 8A–8E). A small open label proof-of-concept clinical trial utilizing fetal VM transplantation to the putamen of PD patients has been performed in Lund, Sweden, with a number of patients experiencing good symptomatic relief (Piccini et al., 1999), but some patients also developed GIDs (Hagell et al., 2002). The brain from one of these patients with a functional graft, who died from other causes 24 years post-transplantation, was harvested for histological analysis. In this brain, we found that the 5-HT6 receptor was very highly expressed inside the TH+ neurons to a much higher extent than found in the host striatum or in other transplanted neurons (Figures 8F–8J). With recent advancements in the differentiation protocols of hESCs into transplantable DA neuroblasts that now approach the fetal-tissue-derived equivalents (Grealish et al., 2014), clinical trials are being envisioned utilizing this cell source in PD. Thus, we evaluated the 5-HT6 expression in transplants originating from the H9 hESC line, differentiated using the novel floor-plate-based protocol (Kirkeby et al., 2012, Kriks et al., 2011) and transplanted into the striatum of immune-compromised nude rats. Also in this case, the 5-HT6 receptor was highly expressed in the hESCs that successfully differentiated into TH+ neurons, but not in any other cells originating from the human transplant or the host striatum (Figures 8K–8O). Together, these data point to the fact that the 5-HT6 receptor may also be important in the clinical setting and that it is not exclusive to fetal-derived DA neurons, but that it is also highly relevant when planning transplantation trials using hESC-derived DA neurons.

## Discussion

Cell transplantation has a unique capacity to restore degenerated neural circuitry. To date, this is a capacity that no alternative therapy in PD (e.g., gene therapy) possesses. However, functional regulation and the issues of GIDs still raise concerns for future clinical trials. In this study, we have for the first time been able to generate a rat model where DA neurons derived from fetal midbrain neuroblasts can be selectively modulated using a bimodal chemogenetic approach. The use of a novel knockin Cre driver line as the source of the DA grafts ensured the selectivity to DA neurons of this regulation. This is, to our knowledge, the first time that cell-type-specific modulation of graft activity is achieved in vivo. Previous studies utilizing optogenetics or DREADDs in hESC- and inducible neuron (iN)-derived transplants, respectively, have both relied on in vitro transduction utilizing pan-neuronal expression of the regulating genes (Dell’Anno et al., 2014, Steinbeck et al., 2015). This is an important technical advancement that makes it possible to dissect the functional contribution of DA neurons contained in the graft, as both fetal and hESC-derived DA transplants contain a broad diversity of both neuronal and nonneuronal cells (Kriks et al., 2011, Thompson et al., 2008).

Using this novel approach, we have been able to make three important discoveries. First, our results show that the ectopic placement of the transplant, i.e., in striatum rather than in the normal location in the SN, while providing significant symptomatic relief, does not recall the full potential of the grafted DA neurons. Through selective activation using chemogenetic receptor stimulation, the therapeutic potential of these transplants was significantly potentiated, providing near-complete recovery in tests of both simple and complex sensorimotor integrative behavior, a level of improvement not reached under baseline conditions. Notably, the functional improvement we observed here following activation of the DA neurons through the cAMP-linked Gs pathway is far greater than has been reported by chemogenetic techniques previously (and no successful activation of transplanted DA neurons using optogenetics has been reported to date) (Dell’Anno et al., 2014). The improvement reported by Dell’Anno from the mouse-fibroblast-derived iDA neurons activated through the Gq pathway was very subtle and similar to the magnitude we observed in the hM3Dq + KORD group (Dell’Anno et al., 2014). Furthermore, we were able to demonstrate that the lesion-induced behavioral deficit is ameliorated by the transplant itself and not through any possible secondary effects. Thus, the reversible inhibition obtained using SalB (in the Gq + KORD rats) reinstated the behavioral deficits in the stepping and cylinder tests. Similar functional reversal has been observed previously when either the entire transplant was physically removed (Björklund et al., 1980) or when the DA neurons of the transplants were selectively killed through injection of 6-OHDA into the graft (Dunnett et al., 1988). A previous report has used optical inhibition of all neurons of the hESC transplant to reinstate a deficit on the corridor test (Steinbeck et al., 2015), but this is the first time that the reversal of motor recovery is achieved through transient silencing of only the transplant-derived DA neurons.

Second, we show that exclusive activation of the DA neurons in the graft through cAMP-linked (Gs-coupled) receptor activation is sufficient to induce GIDs in non-L-DOPA primed parkinsonian rats. While earlier preclinical studies have induced AIMs in DA-grafted, L-DOPA primed rats using d-amphetamine to induce strong release of both serotonin and DA from the host and graft (Lane et al., 2006, Shin et al., 2012), we here for the first time present a GID model that is only dependent on activation of the transplanted DA neurons. Through a comparison of the DA release induced via hM3Dq to that induced via hM3Ds, we could show for the first time that it is not the magnitude of DA release (release amplitude was similar in both cases), but it is the release kinetics that drive the abnormal involuntary movements. Moreover, and in line with previous observations (Carlsson et al., 2007, Lane and Winkler, 2012), the response to apomorphine in the AIMs and rotation tests was markedly reduced in the transplanted groups (Figures 4B and 4D), indicating that the appearance of GIDs is not due to postsynaptic receptor sensitization.

Third, we show that activation of the cAMP-linked, 5-HT6 receptor, expressed on the grafted DA neurons, induces a strong and prolonged DA release in the reinnervated host striatum, and that this stimulation is also sufficient to induce rotational asymmetry and GIDs in the transplanted animals, pointing to a key role of this specific serotonin receptor in the induction of GID. These data point to DA as a key player in the induction of GIDs. Here, we have a unique modulation capacity of DA and have shown that very efficient DA release in itself, as achieved by the hM3Dq DREADD construct, is not sufficient to induce GIDs. The dysplastic DA release as achieved by the rM3Ds is, however, sufficient to induce involuntary movements, both alone and in combination with the hM3Dq DREADD. These findings point to an interesting supersensitivity in grafted DA neurons to changes in cAMP compared to the endogenous nigral DA neurons. This is observed both as a more prolonged DA peak in the in vivo electrochemical recordings and in the dramatically different behavioral changes in response to the 5-HT6 agonist in the grafted animals as compared to the lesioned controls. It is known that activation of the D2 autoreceptor reduces DA release probability through a decrease in cAMP (Weiss et al., 1985). This raises the possibility that the increase in cAMP levels induced by 5-HT6 activation blocks this autoregulatory mechanism in the supersensitive grafted DA neurons, but not in the endogenous DA neurons, resulting in an increased synaptic DA concentration in the transplanted striatum. It is worth noting that there are a number of other receptor subtypes that also regulate cAMP, e.g., muscarinic acetylcholine and adenosine receptors that may also contribute to GIDs. However, we have here shown that 5-HT6 activation alone is sufficient to induce GIDs. Through the studies of the unique patient material and the human-derived tissue transplanted to immune-compromised rats, we have here for the first time shown that the 5-HT6 receptor is highly expressed in transplanted DA neurons and also in transplanted PD patients.

These data are very much in line with the clinical observations by Politis et al. (Politis et al., 2010, Politis et al., 2011). Using PET imaging with the 5-HT transporter imaging ligand ^11^C-DASB, they propose that serotonergic hyperinnervation derived from serotonin neurons contained in the graft is a key contributing factor to GIDs, and they show that silencing of the 5-HT system, using an agonist of the inhibitory 5-HT1A autoreceptor, Buspirone, is efficient in dampening GIDs in grafted patients. These observations point to a GID-inducing signaling cascade where serotonin released from 5-HT terminals acts on the 5-HT6 receptor of cAMP hypersensitive transplanted DA neurons to induce involuntary movements. The clinical relevance of this signaling cascade is further supported by the observation that the 5-HT6 receptor is markedly upregulated in transplanted human midbrain DA neurons, as observed in material obtained from a grafted PD patient, as well as in transplants of human fetal VM tissue and hESC-derived DA neurons transplanted to immune-compromised rats.

This study provides, for the first time, a mechanism through which serotonergic neurotransmission can induce GIDs in grafted PD patients. In patients receiving transplants of fetal VM tissue, the serotonin hyperinnervation caused by 5-HT neurons included in the graft tissue preparation is likely to play an important role. This mechanistic finding should open up more efficient and focused strategies to ameliorate or avoid GIDs in future clinical trials utilizing cell replacement strategies in PD without reducing the therapeutic potential. In addition, our study provides the first demonstration that a combination of chemogenetic receptors can be used as a tool for bimodal regulation of transplanted neurons. This approach may be used clinically to enable fine-tuning of graft activity and thus a more refined approach to restorative therapy, as well as opening up novel routes to investigate the contribution of a transplant to behavioral recovery.

## Experimental Procedures

To evaluate modulatory efficacy of DREADDs in dopaminergic fetal grafts, WT rats (n = 62) received unilateral 6-OHDA lesions to the medial forebrain bundle (MFB), followed by striatal grafting of dopaminergic precursor cells obtained from the VM of TH-Cre rat embryos (n = 38). This was followed by a stereotactic, intraparenchymal injection of an AAV-8 vector containing constructs expressing either hM3Dq + KORD (n = 13), hM3Dq + rM3Ds (n = 12), rM3Ds + KORD (n = 6), or hM4Di + eYFP (n = 6). Two groups of 6-OHDA lesioned animals that did not receive dopaminergic fetal grafts received either no viral vectors (n = 4) or were injected with hM3Dq + rM3Ds viral vectors (n = 4) and used as controls. All animals were evaluated for motor and sensory impairment using a wide array of behavioral tasks. Most hM3Dq + KORD and hM3Dq + rM3Gs animals were sacrificed 18–32 weeks postgrafting, directly following in vivo electrochemical recording, while rM3Ds + KORD and hM4Di + eYFP animals were sacrificed at 40 weeks postgrafting of fetal dopaminergic neurons. Postmortem analysis focused on evaluating surviving dopaminergic neurons within the striatum as well as receptor coexpression in the transplanted cells using immunohistochemistry.

### Animal Research

Adult female Sprague-Dawley rats (225–250 g) were housed in standard laboratory cages with ad libitum access to food and water, under a 12:12 hr dark-light cycle in temperature-controlled rooms. All experimental procedures performed in this study were approved by the regional ethics committee.

### AAV Vectors

DIO-AAV-8 vectors containing the hSyn-rM3Ds-mCherry, hSyn-hM3Dq-HA, hSyn-KORD-IRES-mCitrine, and hSyn-eYFP constructs, flanked 3′ by the woodchuck hepatitis virus posttranscriptional regulatory element (WPRE), were produced by transient transfection in HEK293 cells. Viral titers were determined to range between 4E12 and 1.2E13 using qPCR.

### Preparation of Dopaminergic Fetal Cells

Embryonic day 13.5 TH-Cre heterozygous embryos were removed from the amniotic sac, and the VM from each embryo was carefully dissected and pooled together in ice-cold DMEM/F12 media. Tissue pieces were then incubated with 0.1% trypsin and 0.05% DNase in HBSS for 20 min at 37°C and subsequently mechanically dissociated and engrafted into the striatum of 6-OHDA lesioned WT Sprague-Dawley rats (3 × 2 μL deposits of 4.6E4 viable cells/μl, a total of 275,000 cells).

### Preparation of Mesencephalic DA hESCs

Differentiation of H9 hESCs (WA09, passage 21–45) into dopaminergic progenitors was done as described previously (Kirkeby et al., 2012). Cells were engrafted into 6-OHDA lesioned SD rats using a microtransplantation approach in 2 × 1 μL deposits of 75,000 cells/μL (total of 150,000 cells). Immunosuppressive treatment was administered in the form of daily intraperitoneal (i.p.) injections of cylosporine A (10 mg/kg) beginning 1 day before transplantation.

### Preparation of Primary Human Ventral Midbrain

Human fetal tissue was obtained from 5- to 8-week-old elective terminations of pregnancies and collected with approval of the Swedish national board of health and welfare and in accordance with local ethical guidelines and under informed consent from the donors. Dissection and preparation of the tissue were done as described previously (Björklund et al., 1983, Rath et al., 2013). Fetal tissue was prepared in a semicrude suspension and engrafted into 6-OHDA lesioned SD rats.

### Postmortem Tissue from PD Patient Receiving Fetal Transplant

The preparation of fetal tissue, surgical procedures, and clinical outcome of this patient have been described in detail previously (Hagell et al., 2002, Lindvall et al., 1992, Piccini et al., 1999). Shortly after death, the brain tissue was prepared for specific analyses within the frames of the post-transplantation follow-up study following procedures approved by the Regional Ethical Review Board in Lund. The brain was removed and fixed in 6% buffered formaldehyde solution for 2 months. The basal ganglia were paraffin embedded for subsequent sectioning into 4 μm thick sections.

### Stereotaxic Surgery

Deeply anesthetized rats received a small burr hole through the skull, and the solution containing either 6-OHDA, fetal progenitor cells, or viral vectors was infused unilaterally into the brain using a pulled glass capillary attached to a 25 μL Hamilton syringe. The following coordinates and volumes were utilized: MFB lesion, 3 μL of 6-OHDA at AP = −4.4, ML = −1.1, and DV −7.8 with infusion rate of 0.3 μL/min (Figure S1A). For DA grafting, 3 × 2 μL at three sites (defined as mm from bregma in AP and ML and from dura in DV): (1) AP = +1.8, ML = −2.5, and DV = −4.5; (2) AP = +0.6, ML = −2.0, and DV = −4.5; and (3) AP = +0.6, ML = −3.2, and DV = −4.5, with an infusion rate of 0.4 μL/min. For AAV-8 viral vectors injected into the striatum: 3 μL in the center of the three grafts with two deposits, AP = +1.0, ML = −2.6, and DV = −4.5 and −3.5, with an infusion rate of 0.4 μL/min.

### Tissue Preparation and Immunohistochemistry

Rat brains were fixed in situ using transcardial perfusion of 4% paraformaldehyde (pH 7.4) in 0.1 M phosphate buffer and cryoprotected in buffered sucrose (25%). They were then cut into coronal or axial sections with a thickness of 35 μm and 45 μm, respectively. Human tissue from grafted putamen was fixed postmortem in 6% formalin for 1 month and then embedded in paraffin. Paraffin-embedded tissue was then cut into 4 μm thick coronal sections. Using a standard free-floating immunohistochemistry protocol, the following antibodies were utilized: anti-TH (Millipore, Cat #AB152, RRID: AB_390204, 1:1,000), anti-5-HT6 (Novus, Cat #NBP1-46557, RRID: AB_10009833, 1:1,000), anti 5-HT6 (Santa Cruz, Cat #sc-26668, RRID: AB_2280074, 1:1,000), anti-SERT (Millipore, Cat #MAB1564, RRID: AB_94220, 1:1,000), anti-human-NCAM (Santa Cruz, Cat #sc-106, RRID: AB_627128, 1:1,000), anti-HA-tag (Covance, Cat #MMS-101R-200, RRID: AB_10064220, 1:2,000), anti-mCherry (LifeSpan Biosciences, Cat #LS-C204207, 1:1,000), and anti-GFP (Abcam, Cat #ab13970, RRID: AB_300798, 1:20,000). Biotinylated secondary antibodies were utilized for DAB immunohistochemistry and amplified by Vectorlabs ABC kit. For immunofluorescence, Alexa-conjugated secondary antibodies were utilized.

### Behavior Tests

Rotational locomotion was quantified in automated rotometer bowls (AccuScan Instruments, Inc.), where the rats were allowed to habituate in the rotometer for 10 min prior to drug injection. As described in Figure 1A, the animals’ rotational behavior was quantified after injection of d-amphetamine (2.5 mg/kg, i.p.), apomorphine (0.05 mg/kg, subcutaneously [s.c.]), CNO (3 or 10 mg/kg, s.c.), and 5-HT receptor agonists (5–20 mg/kg, s.c.).

Forelimb akinesia was assessed using the side-stepping test (Olsson et al., 1995), where forelimb adjusting steps are quantified over a total length of 90 cm.

Forelimb asymmetry in exploratory behavior was assessed using the cylinder task (Björklund et al., 2010), where the rat is placed in a glass cylinder (20 cm in diameter) and at least 30 touches between paw and the walls are recorded with a digital video camera for post hoc analysis.

Lateralized sensorimotor neglect was characterized in three different tests: the corridor task (Dowd et al., 2005), disengage task, and sensorimotor orientation (Mandel et al., 1990). In the corridor task, the rats were placed inside an opaque plastic corridor with ten pairs of food bowls, each filled with 5–10 sugar pellets, where the retrievals are recorded relative to the rats’ visual fields.

In the disengage task, the animals were placed on an elevated platform and given pieces of milk chocolate (Mandel et al., 1990, Winkler et al., 2000), after which the perioral region beneath the vibrissae on each side of the head was repeatedly touched with the use of a wooden probe. Latency to respond was then scored between 1 s (direct response) and 180 s (no response).

In all tests, once a stable baseline was achieved the animals were injected with CNO (3 or 10 mg/kg, s.c.), SalB (10 mg/kg, s.c.), or a vehicle and tested at 60 or 15 min postinjection, respectively. The researcher was blinded to both animal group and treatment.

### Abnormal Involuntary Movements

The rats were assessed for abnormal involuntary movements using a well-characterized rating scale through rater-blinded assessment in empty transparent cages (Lundblad et al., 2002). After habituation for 10 min, animals were injected with CNO (3 mg/kg, s.c.) or ST-1936 (20 mg/kg, i.p). The animals were scored for a total of 2 hr postinjection, with each animal being scored every 20 min. Animals were scored for limb, axial, and orolingual abnormal involuntary movements as well as general locomotion.

### In Vivo Electrochemistry

In vivo chronoamperometry was performed using FAST-16 mk II hardware coupled to nafion-coated carbon fiber electrodes (∅ 30 μm, L 150 μm) according to (Hoffman and Gerhardt, 1998). Local application of CNO and KCl was made by pulse pressure ejection from glass capillaries mounted ∼50–100 μm from the electrode tip.

### Laser-Scanning Confocal Microscopy

All LSM (laser-scanning confocal microscopy) was conducted using a Leica SP8 setup where images were captured using a HyD detector and always with the lasers activated in sequential mode using solid-state lasers at wavelengths of 405, 488, 552, and 650 nm (a pinhole of 1 AU).

### Statistics

Statistical tests were performed in SPSS version 23 and include Bonferroni corrected paired Student’s t test and one-way ANOVA or two-way mixed-model repeated-measures ANOVA when three or more groups/states/time points are compared, followed by Levene’s homogeneity test. The locus of effect was probed using either Dunnett’s T3 or Tukey’s least significant difference test as appropriate based on the outcome of the Levene’s test. Nonparametric data were analyzed using Kruskal-Wallis test followed by Bonferroni corrected all-pair comparison using the Mann-Whitney U test. Unless otherwise noted, data in figures are presented as the arithmetic mean with the SEM.

## Author Contributions

T.B., P.A.-K., M.L., and A.H. designed the experiment; P.A.-K. and B.M. performed the fetal dissections and stereotactic surgeries; P.A.-K., B.M., and G.W. performed the behavioral analyses; A.H. performed the in vivo recordings, A.H. and M.L. analyzed the electrochemistry data; P.A.-K. performed the postmortem analyses; P.A.-K., A.H., M.P., and T.B. wrote the manuscript.

## Supplemental Information

Document S1. Supplemental Experimental Procedures and Figures S1–S6

Table S1. Collected Quantitative Data from In Vivo Electrochemical Recordings Relates to Figures 2, 5 and S6. This spreadsheet contains raw values from the electrochemical recordings for each animal with calculation of peak amplitude (Amp), the rise time (Trise), and re-uptake times.

Movie S1. Representative Video Recording of Abnormal Involuntary Movements Induced through Selective Increase of cAMP in the Fetal DA Transplant Relates to Figure 4. The movie shows one representative animal from the hM3Dq + rM3Ds DREADD group. The movies are recorded 24 weeks following fetal grafting (15 weeks post-AAV injection). On 2 consecutive days, the animal was recorded in the empty cage after 3 mg/kg CNO (first day, left) or saline (second day, right). The two movies are time synced to display the same time point postinjection. Time points selected are representative for the developments of the different abnormal involuntary movements observed. The lesion-control animals develop only a transient locomotor increase, and the TH-TX hM3Dq + KORD animals displayed no change in behavior after CNO administration.

Document S2. Article plus Supplemental Information
