# Supplementary material for: DREADD Modulation of Transplanted DA Neurons Reveals a Novel Parkinsonian Dyskinesia Mechanism Mediated by the Serotonin 5-HT6 Receptor
Source: Neuron. 2016 Jun 1;90(5):955–68. doi: 10.1016/j.neuron.2016.04.017 (PMC4893163; doi:10.1016/j.neuron.2016.04.017)
Supplement: Document S1. Supplemental Experimental Procedures and Figures S1–S6 [file mmc1.pdf]

Neuron, Volume *90*

Supplemental Information

DREADD Modulation of Transplanted DA Neurons Reveals a Novel Parkinsonian Dyskinesia Mechanism Mediated by the Serotonin 5-HT6 Receptor

Patrick Aldrin-Kirk, Andreas Heuer, Gang Wang, Bengt Mattsson, Martin Lundblad, Malin Parmar, and Tomas Björklund

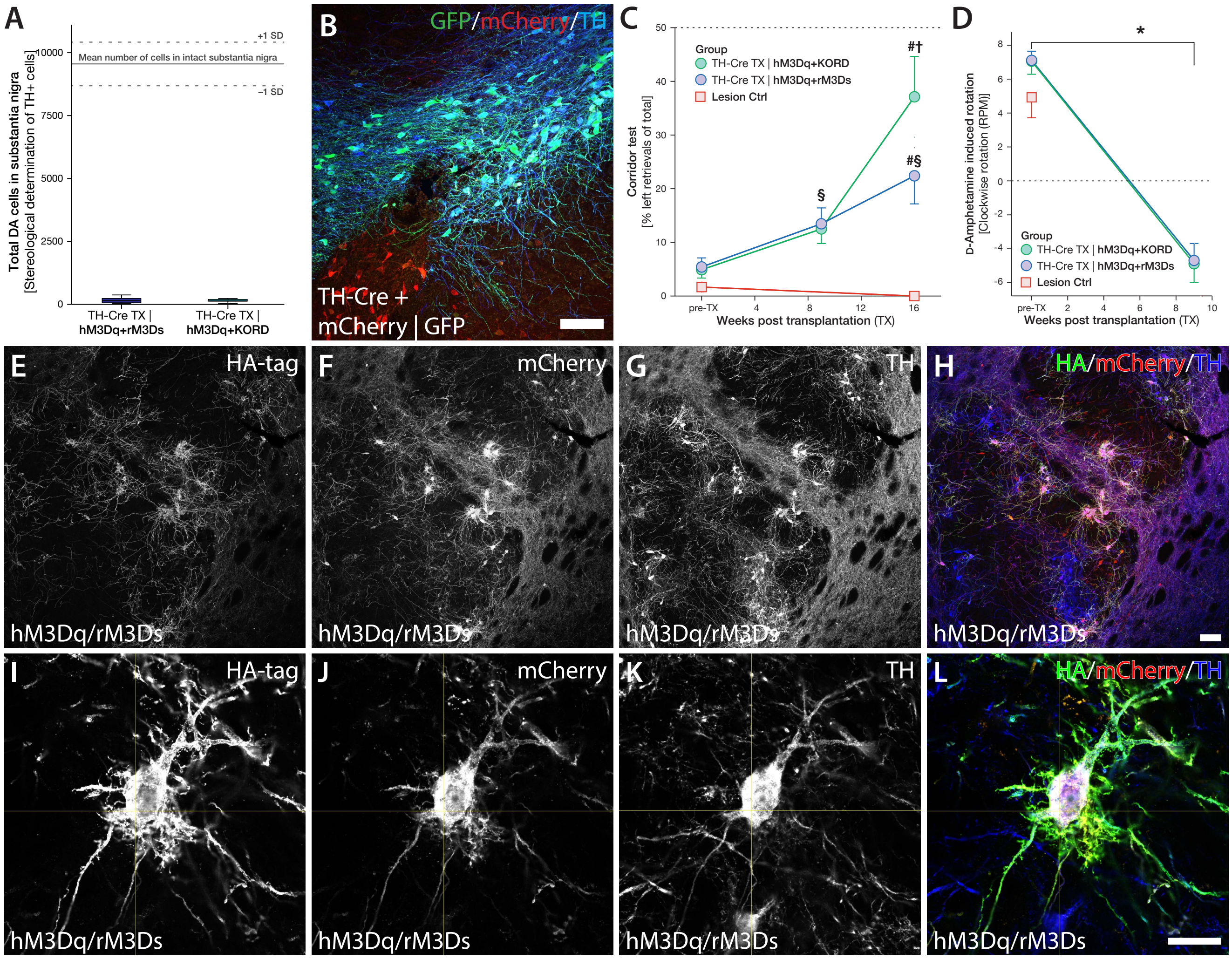

Supplemental figure S1 | relates to Figure 1

(A) Validation of complete lesion of midbrain dopaminergic neurons following 6-OHDA neurotoxin injection into the medial forebrain bundle, compared to mean number of dopaminergic neurons contralateral to the lesion (solid line +/- 1SD). (B) SN dopaminergic neurons virally transduced with a fluorescence shifting AAV-8 mCherry-DIO-GFP vector in which non Cre expressing neurons are labeled with mCherry and Cre expressing neurons were labeled with GFP. Staining for TH (blue) mCherry (red) and GFP (green) showed that only TH positive SN neurons were expressing GFP, while surrounding, non-dopaminergic neurons are highly positive for mCherry, confirming that it is the high specificity of Cre expression to dopaminergic neurons that allows for the selective expression and not a difference in vector tropism. (C) Corridor task at baseline post lesion (pre-TX), 8 wks following fetal grafting at 8 and 4 wks post AAV injection (16 wks) for hM3Dq + rM3Ds (green), hM3Dq + KORD(blue) and lesion control animals (red). (D) Validation of dopaminergic grafting using amphetamine-induced rotational behavior. Successful grafting of dopaminergic neurons caused both hM3Dq + rM3Ds (blue) and hM3Dq + KORD (green) animals to overcompensate from ipsilateral to contralateral rotational behavior in response to amphetamine. (E-H) Immunofluorescence overview of a dopaminergic fetal graft using laser-scanning confocal microscopy, stained for HA-tag (E), mCherry (F), TH (G) with a pseudo-colored overlay (H). (I-J) Morphology of a single transduced grafted dopaminergic neuron, stained for HA-tag (I), mCherry (J), TH (K) with an overlay (L). All values reported as arithmetic mean ± 1 SEM. * $p < 0.05$ in Bonferroni corrected paired Student's t-test; # Significantly different from Ctrl ($p < 0.05$) in repeated measures ANOVA, followed by Bonferroni corrected one-way ANOVA followed by Dunnett's T3 post hoc test as Levene's Homogeneity test failed; § Significantly different from pre-TX in repeated measures ANOVA, followed by Bonferroni corrected paired Student's t-test; † Significantly different from 9-wks post-TX in repeated measures ANOVA followed by Bonferroni corrected paired Student's t-test. Scale bar in B and L represent 100µm and 25 µm respectively.

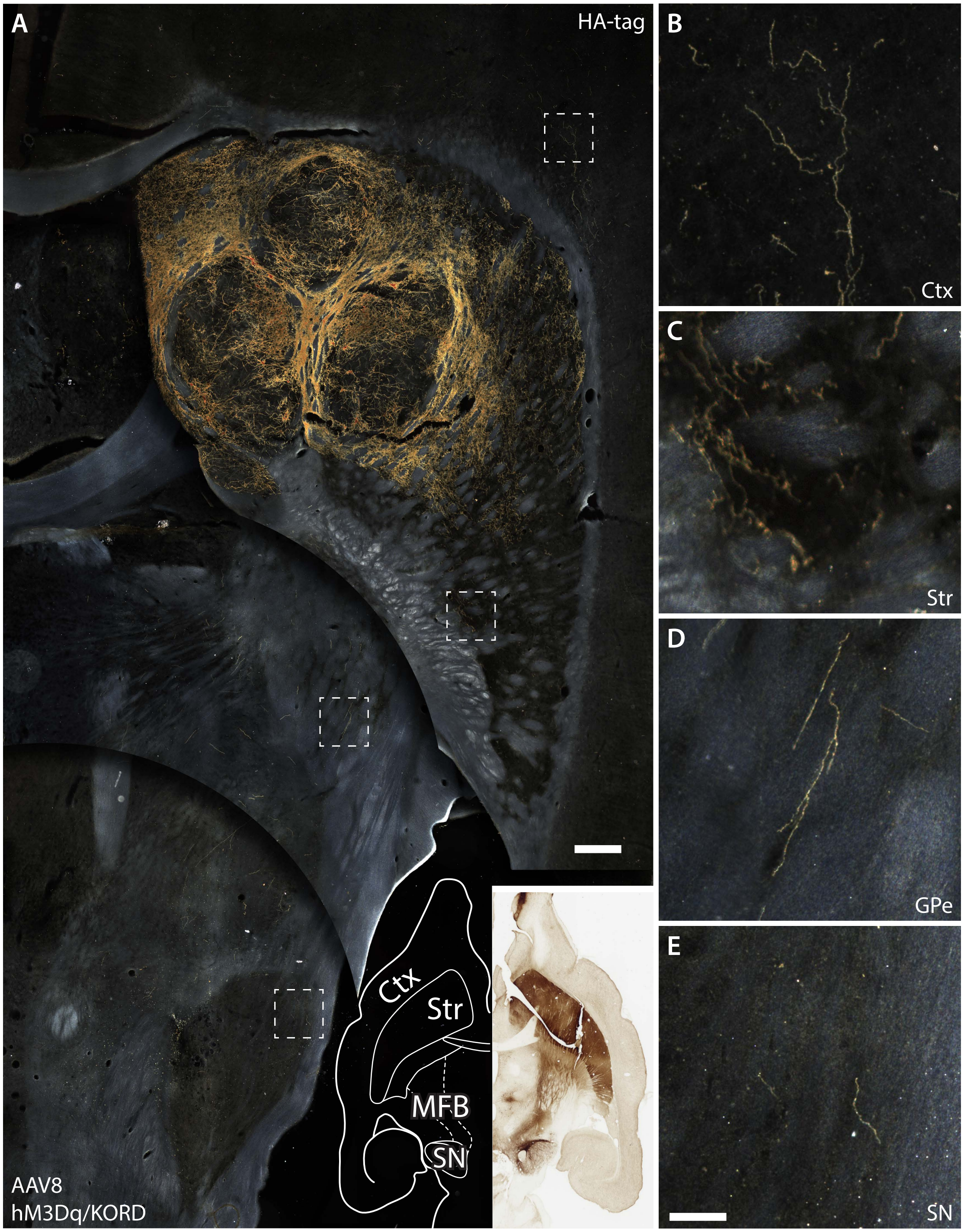

Supplemental figure S2 | relates to Figure 1

Histological analysis of dopaminergic innervation originating from transduced HA-tagged grafted dopaminergic neurons visualized as a montage overview (A) captured through dark field microscopy. HA tagged projections were found in the cerebral cortex (B), caudal striatum (C), along the medial forebrain bundle in the Globus Pallidus (D) and in the Substantia Nigra (E). Insert in A shows a horizontal section of an intact DA system visualized through DAB stain against TH. Scale bar in A and E represent 250µm and 50 µm respectively.

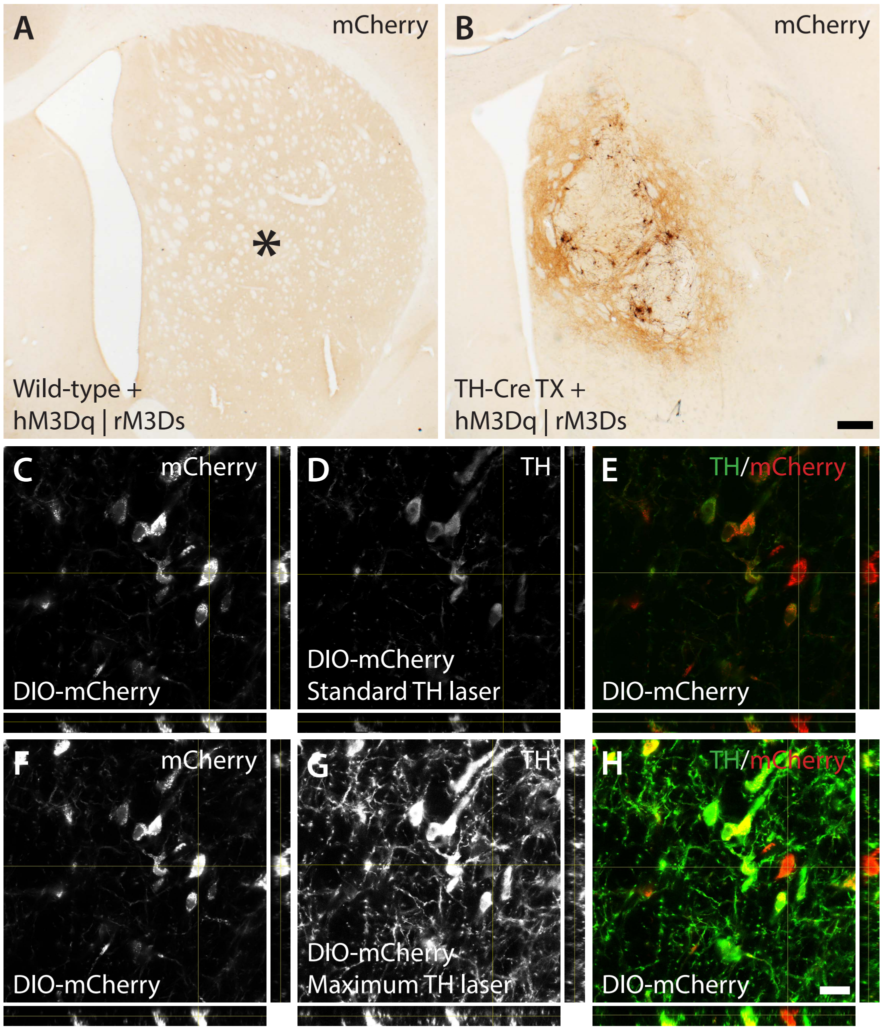

Supplemental figure S3 | Relates to Figure 2

(A-B) Expression of Double-floxed Inverted Orientation (DIO) DIO-hM3Dq and DIO-rM3Ds DREADDs, following viral infusion into the striatum of wild-type animals and animals grafted with dopaminergic fetal tissue from TH-Cre transgenic rats. * Denotes position of viral infusion. These Cre-inducible viral vectors displayed high selectivity to grafted tissue and no significant expression was detected in the wild type striatum, suggesting a highly selective expression to Cre expressing tissue. (C-H) Laser scanning confocal (LSM) microscopy of midbrain dopaminergic neurons, virally transduced with AAV-8 DIO-mCherry and stained for TH (green) and mCherry (red) in the VTA. LSM z-stack of mCherry positive neurons (red) observed to have undetectable TH expression (green) under normal laser power conditions. Although expression of virally transduced transgenes was very specific in the SN a sub-population of neurons in the VTA displayed undetectable TH expression (C-E). Laser scanning confocal microscopy z-stack of the same mCherry positive neuron (red) was observed to have low level detectable TH expression (green) under high laser power conditions, suggesting that VTA dopaminergic neurons may have a heterogeneous expression of TH, some at very low levels (F-H). Scale bar in B and H represent 200μm and 25 μm respectively.

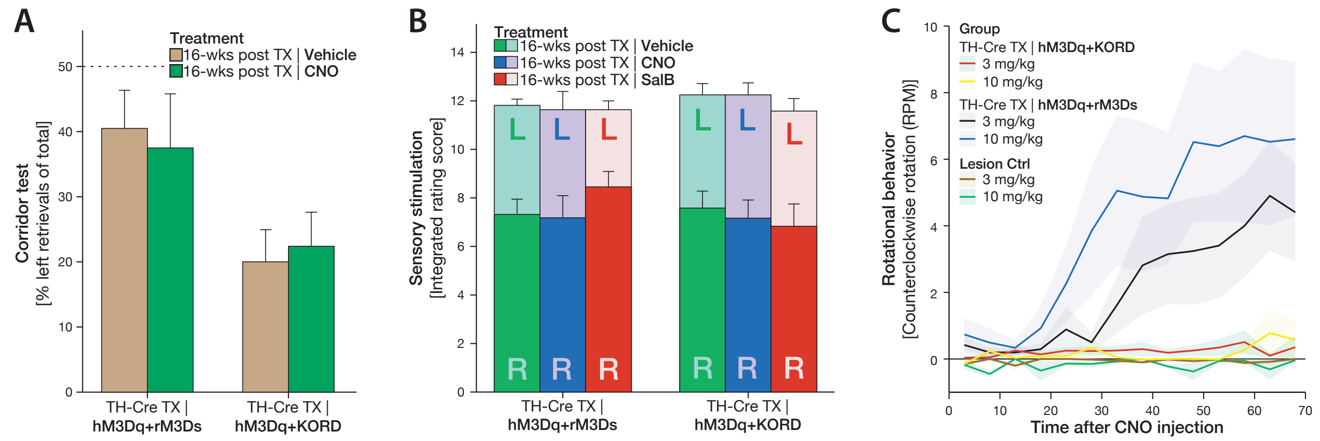

Supplemental figure S4 | Relates to Figure 3

(A) Corridor task at 4 wks post AAV injection (16 wks) following CNO treatment (green) and vehicle (tan) for both hM3Dq + rM3Ds and hM3Dq + KORD animals. (B) Response latency to sensory stimulation on contralateral [L] and ipsilateral [R] sides of the body following treatment with vehicle (green), CNO (blue), and SalB (red). (C) CNO treatment induced rotational behavior in hM3Dq + rM3Ds animals in a dose dependent manner, 3mg/kg (black) and 10 mg/mg (blue). CNO did not induce any rotational behavior in hM3Dq + KORD (red and yellow) or lesion controls (brown and green) at either 3mg/kg or 10mg/kg, respectively. All values reported as arithmetic mean ± 1 SEM.

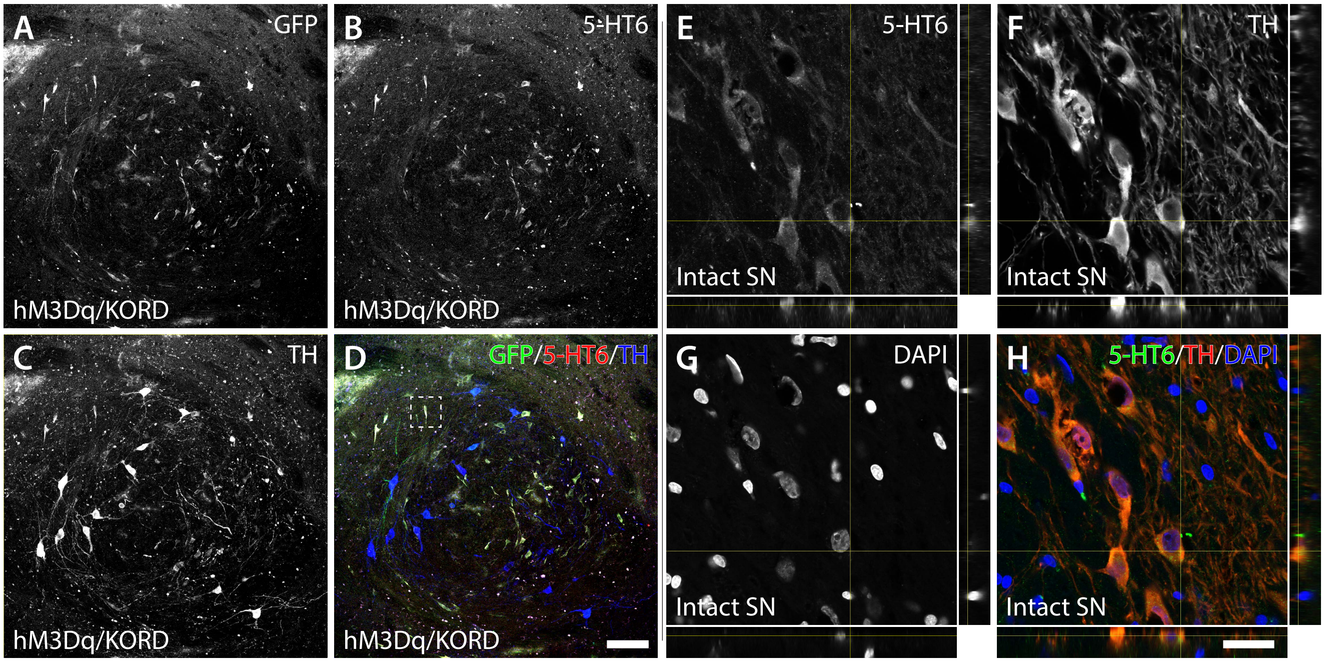

Supplemental figure S5 | Relates to Figure 5

(A-D) Overview of a dopaminergic fetal graft stained for GFP (A), 5-HT6 (B), TH (C) with an overlay (D). (E-H) Immunofluorescence z-stack showing 5-HT6 expression in nigral dopaminergic neurons using confocal microscopy with staining for 5HT-6 (E), TH (F) and DAPI (G) with an overlay (H). All values reported as arithmetic mean ± 1 SEM. * significantly different to the Lesion Ctrl group, $p < 0.05$ using one-way ANOVA followed by Dunnett's T3 post hoc (as the Levene's Homogeneity test was significant). Scale bar in D and H represent 100µm and 25 µm respectively.

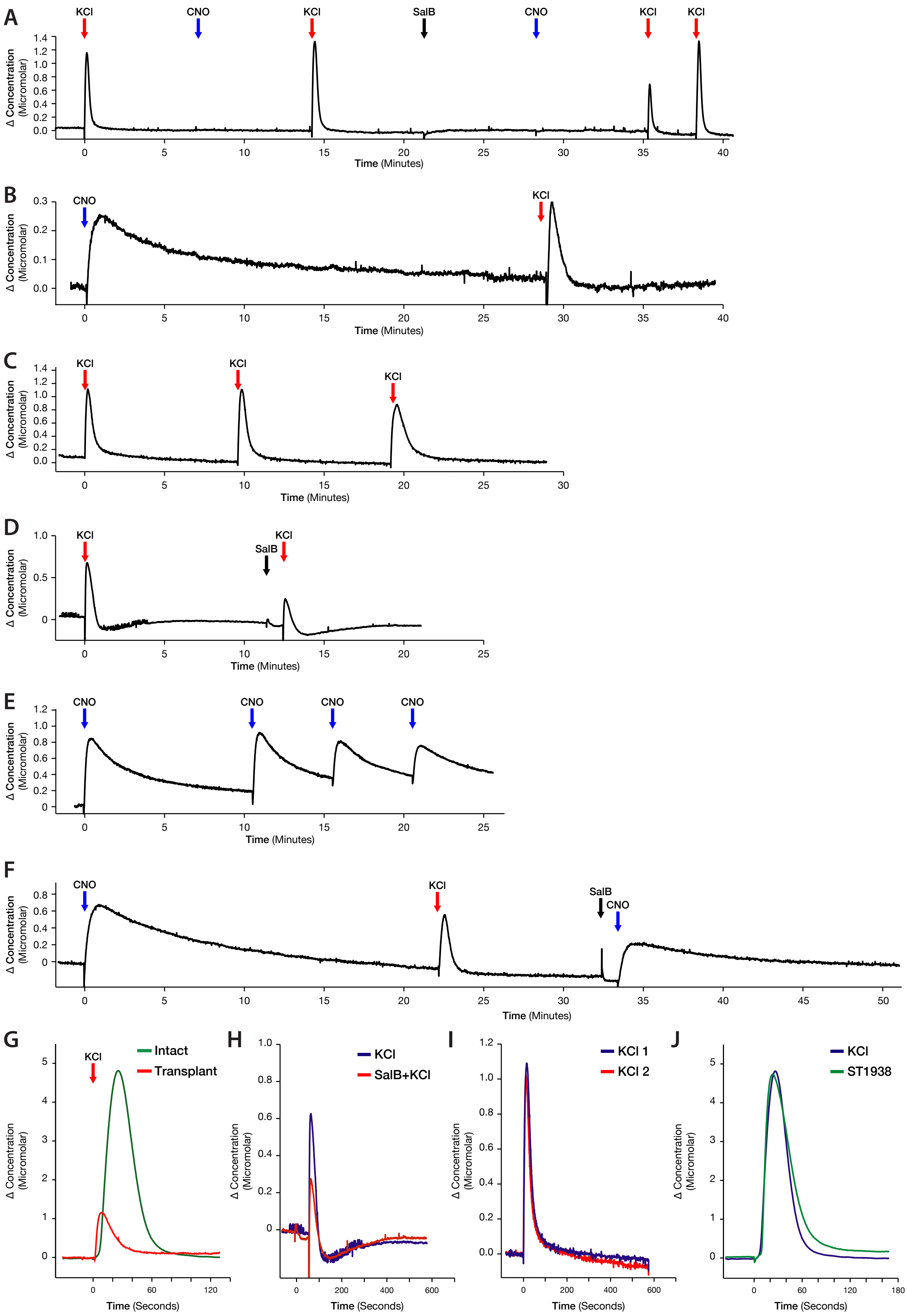
A
KCl
CNO
KCl
SalB
CNO
KCl
KCl
Δ Concentration (Micromolar)
Time (Minutes)
B
CNO
KCl
Δ Concentration (Micromolar)
Time (Minutes)
C
KCl
KCl
KCl
Δ Concentration (Micromolar)
Time (Minutes)
D
KCl
SalB
KCl
Δ Concentration (Micromolar)
Time (Minutes)
E
CNO
CNO
CNO
CNO
Δ Concentration (Micromolar)
Time (Minutes)
F
CNO
KCl
SalB
CNO
Δ Concentration (Micromolar)
Time (Minutes)
G
KCl
Intact
Transplant
Δ Concentration (Micromolar)
Time (Seconds)
H
KCl
SalB+KCl
Δ Concentration (Micromolar)
Time (Seconds)
I
KCl 1
KCl 2
Δ Concentration (Micromolar)
Time (Seconds)
J
KCl
ST1938
Δ Concentration (Micromolar)
Time (Seconds)

Supplemental figure S6 | Relates to Figure 5

(A) Control measurements in the intact striatum using electrochemical chronoamperometric recordings of DA release. Local KCl administration (red arrow) evoked strong DA release while neither CNO (Blue arrow) nor SalB (black arrow) administration resulted in any measurable increase in extracellular DA. (B) Recordings of local CNO administration (blue arrow) inducing DA release in a DIO-rM3Dq + KORD animal, which was repeatable with local KCl administration (red arrow) once a stable baseline had again been obtained. Note that the CNO evoked DA release takes about 25 minutes to return to baseline. (C,I) representative sample traces of local administration of KCl showing repeatability of the system to evoke similar levels of DA release following a return to baseline levels with a 7-minute interval being sufficient to be able to elicit a full DA release. Local administration of SalB (black arrow) 10" before KCL injection attenuated the KCl response (D and H) in hM3Dq + KORD animals. (E) CNO administration (blue arrow) to hM3Dq + KORD animals also induced a repeatable DA release. Note that the first CNO response in the sample trace was recorded for 10 minutes before "pushing" the system in 3 successive CNO injections with a 5' interval. (F) Similarly to KCl evoked DA release, SalB (black arrow) attenuated the DA release evoked by CNO (blue arrow) in hM3Dq + KORD animals. (G) The DA release induced by KCl, measured in the innervated area (red), was robust but uniformly lower than that measured in the intact striatum (green). (J) In the intact striatum, the 5-HT6 receptor agonist ST1936 induced a DA release identical to KCl induced release (blue) with regards to amplitude and kinetics.

Supplemental movie 1 | Relates to Figure 4

The movie shows one representative animal from the hM3Dq + rM3Ds DREADD group. The movies are recorded 24 wks following fetal grafting (15 wks post AAV injection). On two consecutive days, the animal was recorded in the empty cage after 3 mg/kg CNO (first day, left) or saline (second day, right). The two movies are time-synced to display the same time-point post injection. Time-points selected are representative for the developments of the different abnormal involuntary movements observed. The lesion-ctrl animals develops only a transient locomotor increase and the TH-TX hM3Dq + KORD animals displayed no change in behavior after CNO administration.

Supplemental spreadsheet 1 | Relates to Figures 2 & 5 and Supplemental figure S6

This spreadsheet contains raw values from the electrochemical recordings for each animal with calculation of peak amplitude (Amp), the rise time (Trise) and re-uptake times .

SUPPLEMENTAL METHODS

Animal Research

Female Sprague Dawley rats (225-250g) were purchased from Charles River (Germany) and were housed with free access to food and water under a 12 hours light/12 hours dark cycle in a temperature-controlled room. All experimental procedures performed in this study were approved by the Ethical Committee for Use of Laboratory Animals in the Lund-Malmö region.

AAV vector production

Double-floxed Inverted Orientation (DIO) AAV-8 vectors, containing the hSyn-hM3Dq-HA, hSyn-KORD-IRES-mCitrine, hSyn-eYFP and hSyn-mCherry-flex-GFP constructs, were produced by dual-plasmid, calcium precipitation mediated transient transfection of HEK-293 cells and purified by iodixanol gradient centrifugation and anion exchange chromatography as described previously (Zolotukhin *et al.,* 2002). The sequences were flanked 3' UTR by the Woodchuck hepatitis virus post-transcriptional regulatory element (WPRE) and terminated with a SV40 derived poly-adenylation sequence, in order to increase mRNA cytosolic half-life. Viral titers were quantified via qPCR, with primers recognizing the AAV ITR sequences yielding working titers of 8.0E12 and 1.2E13 GC/ml for the AAV-8-hM3Dq and AAV-8-KORD respectively. The AAV-8-hSyn-rM3Ds-mCherry viral vector used was purchased from UNC vector core, Chapel Hill with a titer of 4.0E12.

Preparation of dopaminergic fetal cells

Male TH-Cre homozygote Sprague-Dawley males (supplied by SAGE labs, now Horizon Discovery, TGRA8400) (Brown *et al.,* 2013) were time mated with wild type Sprague-Dawley female rats (supplied by Charles river). At E13.5, the pregnant females were anesthetized, using a CO_2 chamber and the embryonic sacs were carefully removed and transferred to ice-cold DMEM/F12 media (Gibco, Life Technologies). Following the removal of the embryo from the embryonic sac, the ventral mesencephalon was carefully dissected from each fetus and pooled together in ice-cold DMEM/F12 media. The culture media was then removed and incubated with 0,1 % trypsin (Gibco, Life Technologies) and 0,05 % DNAse (Qiagen) in HBSS (Gibco, Life Technologies) for 20 minutes at 37° C. The trypsin solution was then washed away (x3) and replaced with the HBSS/DNAse solution. The cells were then dissociated by mechanically pipetting the solution until a milky solution with no obvious pieces of tissue remained. Following centrifugation at 600 g for 5 minutes the cells were re-suspended in in HBSS/DNAse and cell count / viability quantified using trypan blue. Cell concentration was then diluted to 4.6E4 viable cells/µl. A total of 275 000 cells were then grafted into the striatum of 6-OHDA lesioned wild type Sprague-Dawley rats along three needle tracts.

Differentiation and transplantation of mesDA hESCs

hESC H9 (WA09, passage 21-45) cells were maintained and passaged weekly on mouse embryonic fibroblasts in DMEM/F12, 20% KSR, 0.05 mM 2-mercaptoethanol 0.5% Pen./Strep. and 10 ng/ml FGF-2 and differentiated into ventral midbrain fate from cells according to the previously described protocol (Grealish *et al.,* 2014; Kirkeby *et al.,* 2012). Following aspiration of the culture media, hESC were washed in PBS and dissociated through treatment of 0.5 mM EDTA. Cells were then replated in differentiation medium: NIM, Y-27632 (10 µM), SB431542 (10 µM), noggin (100 ng/ml), SHH-C24II (20ng/ml) and CT99021 (0.8 µM) on untreated culture plates, allowing formation of embryonic bodies (EBs). For differentiation day 0-2 ROCK inhibitor was added to the culture medium in order to increase EB formation in the culture. On differentiation day 4 EB culture was transferred to PO/lam/FN coated plates in NPM medium with SB431542 (10 µM) and noggin (100 ng/ml) followed by plain NPM medium on day 9. On day 11 the culture was re-plated in NDM with BDNF (20 ng/ml), GDNF (10 ng/ml) and AA (0.2 mM). From day 14 the culture was changed and maintained in NDM with BDNF (20 ng/ml), GDNF (10 ng/ml), ascorbic acid (0.2 mM), db-cAMP (500 µM) and DAPT (1 µM) until the cells reached full neuronal maturation. For transplantation, cells were dissociated with accutase and re-suspended in HBSS and DNAse (0.05 %). The cells were then grafted into the striatum of 6-OHDA lesioned SD rats at the following coordinates relative to the bregma: AP: +0.8; ML: +3.0; DV: -4.0; tooth bar: -3.3. 2µl of the cell suspension (75000 cells/µl) was deposited at the infusion site. Immunosuppressive treatment was administered in the form of daily i.p. injections of Cyclosporine A (10mg/kg) beginning 1 day before transplantation.

Preparation and transplantation of primary human ventral midbrain

Human fetal tissue was obtained from 5-8 week old elective terminations of pregnancies and collected with approval of the Swedish national board of health and welfare and in accordance with local ethical guidelines and under informed consent from the donors. Dissection and preparation of the tissue was done as previously described by (Grealish *et al.,* 2014). Tissue from two fetal VMs (6 and 7.5 weeks p.c.) were cut into smaller pieces and incubated for 20 min at 37°C in DMEM with 0.1 % trypsin and 0.05% DNAse. The tissue was then mechanically dissociated by careful repeated trituration starting with a 1 ml pipette followed by use of a 200 µl pipette in order to achieve a single-cell suspension. Cells were then centrifuged at 600 rpm for 5 minutes, the supernatant removed and the cell pellet re-suspended in DMEM with 0.05% DNAse. The cell suspension was then transplanted into 4 separate site at the following coordinates (relative to bregma) within the rat striatum: AP: +1.2; ML: -2.6; DV: -5.0 (3 µl) and -4.0 (3 µl); and AP: +0.5; ML: -3.0; DV: -5.0 (3 µl) and -4.0 (3 µl); tooth bar -2.4.

Post-mortem tissue from PD patient receiving fetal transplant | The preparation of fetal tissue, surgical procedure and clinical outcome of this patient has been described in detail previously (Hagell *et al.,* 2002; Lindvall *et al.,* 1992; Piccini *et al.,* 1999). Briefly, immunosuppressive treatment was initiated 2 days before surgery and continued for 1-year post transplantation. Dissociated ventral mesencephalic tissue (sourced and prepared as above) was implanted into the anterior, middle and posterior part of the putamen contralateral to the most affected limb. Shortly after death, the brain tissue was prepared for specific analyses within the frames of the post transplantation follow-up study following procedures approved by the Regional Ethical Review Board in Lund. The brain was removed and fixed in 6% buffered formaldehyde solution for 2 months. The basal ganglia were paraffin embedded for subsequent sectioning into 4µm thick sections.

Stereotaxic surgery

Prior to all stereotaxic surgery procedures, rats were deeply anesthetized with fentanyl-dormitor (i.p.) (Apoteksbolaget) and placed in the stereotactic frame with the tooth bar individually adjusted for flat skull (bregma-lambda; TB: -3 to -4 mm). Targeting coordinates for all stereotactic infusions were identified relative to the bregma. A small hole was drilled through the skull and the solution containing either 6-OHDA, fetal progenitor cells or viral vectors were infused unilaterally into the brain. Injections were performed using a pulled glass capillary (60–80 µm i.d. and 120–160 µm o.d.) attached to a 25µl Hamilton syringe connected to an automated infusion pump system. For unilateral dopaminergic lesions, 3µl of 6-OHDA (Cl-salt, Sigma-Aldrich) [14 µg free base in ascorbate-saline (0.02%) injected at a concentration of 3.5 µg/µl] was infused into the medial forebrain bundle (MFB) AP= -4.4; ML = -1.1; DV -7.8 with an infusion rate of 0,3µl/min (Ungerstedt and Arbuthnott, 1970). Grafting of fetal dopaminergic progenitor cells were infused at three sites within the striatum (3x2µl) (1) AP= +1.8; ML= -2.5; DV= -4.5, (2) AP= +0.6; ML= -2.0; DV= -4.5, (3) AP= +0.6; ML= -3.2; DV= -4.5, with an infusion rate of 0.4µl/min. Infusion of AAV-8 viral vectors into the striatum used 3µl of hM3Dq + rM3Ds or 3µl of hM3Dq + KORD and was placed in the center of the three grafts with two deposits. AP= +1.0; ML= -2.6; DV= -4.5; DV=-3.5, with an infusion rate of 0.4µl/min. AAV-8 viral vectors infusion into the ventral midbrain: 3µl of viral vector at AP= -5.3; ML= -1.7; DV= -7.2, with an infusion rate of 0.2µl/min. The capillary was left in position for two minute before retraction, following all infusions.

Tissue preparation and immunohistochemistry

Animals were sacrificed between 18-20 weeks post grafting following amperometry measurements by sodium pentobarbital overdose (Apoteksbolaget) and trans-cardially perfused with 150 ml physiological saline solution followed by 250 ml of freshly prepared, ice-cold, 4% paraformaldehyde (PFA) in 0.1 M phosphate buffer (pH = 7.4). The brains were removed and post-fixed for 2 hours in ice-cold PFA before storing in 25% buffered sucrose for cryoprotection for at least 24 hours until further processing. The brains were then cut into coronal and axial sections with a thickness of 35µm and 45µm respectively, using a sliding microtome (HM 450, Thermo Scientific). The brain sections were collected as 1 in 8 series and stored in anti-freeze solution (0.5M sodium phosphate buffer, 30% glycerol and 30% ethylene glycol) at −20°C until further processing. For immunohistochemical analysis, tissue sections were washed (3x) with TBS (pH 7.4) and incubated for one

hour in 3 % H2O2 in 0,5 % TBS Triton solution in order to quench endogenous peroxidase activity and to increase tissue permeability. Following another washing step, the sections were blocked in 5 % bovine serum and incubated for one hour and subsequently incubated with primary monoclonal antibodies overnight. Dopaminergic neurons were identified through staining for tyrosine hydroxylase (Rabbit anti-TH, Millipore Cat# AB152 RRID:AB_390204, 1:1000) and 5-HT6 expressing neurons were identified through staining of 5-HT6 receptor (mouse anti-5-HT6, Novus Cat# NBP1-46557 RRID:AB_10009833, 1:1000 or rabbit anti 5-HT6, Santa Cruz Biotechnology Cat# sc-26668 RRID:AB_2280074, 1:1000). In order to evaluate transduction and expression efficacy, hM3Dq expressing neurons were stained for the HA-tag (mouse anti-HA, Covance Research Products Inc Cat# MMS-101R-200 RRID:AB_10064220, 1:2000), rM3Ds for mCherry (goat anti- mCherry, LifeSpan Biosciences Cat#LS-C204207, 1:1000) and KORD for GFP (chicken anti-GFP, Abcam Cat# ab13970 RRID:AB_300798, 1:20000). Grafted human tissue was evaluated by staining for hNCAM (mouse anti-hNCAM, Santa Cruz Biotechnology Cat# sc-106 RRID:AB_627128, 1:1000). Following overnight incubation, the primary antibody was first washed away using TBS (x3) and then incubated with secondary antibodies for two hours. For 3, 30-diaminobenzidine (DAB) immunohistochemistry, biotinylated anti-mouse (Vector Laboratories Cat# BA-2001 RRID:AB_2336180, 1:250), anti-rabbit (Vector Laboratories Cat# BA-1000 RRID:AB_2313606, 1:250), anti-goat (Jackson ImmunoResearch Labs Cat# 705-065-147 RRID:AB_2340397, 1:250) and anti-chicken (Vector Laboratories Cat# BA-9010 RRID:AB_2336114, 1:250) secondary antibodies were used. For immunofluorescence, Alexa fluor conjugated anti-rabbit (Jackson ImmunoResearch Labs Cat# 711-165-152 RRID:AB_2307443, 1:250), anti-rabbit (Jackson ImmunoResearch Labs Cat# 711-606-152 RRID:AB_2340625, 1:250) anti-mouse (Jackson ImmunoResearch Labs Cat# 715-605-151 RRID:AB_2340863, 1:250), anti-mouse (Jackson ImmunoResearch Labs Cat# 715-545-150 RRID:AB_2340846, 1:250) anti-goat, (Molecular Probes Cat# A11056 RRID:AB_142628, 1:250) and anti-chicken (Thermo Fisher Scientific Cat# A11039 RRID:AB_10563770, 1:250) antibodies were used. For DAB immunohistochemistry, the ABC-kit (Vectorlabs) was used following incubation of the secondary antibody to amplify the staining intensity through streptavidin-peroxidase conjugation and followed by a DAB in 0.01% H_2O_2 color reaction.

Rotational locomotion

Rotational locomotion was used to evaluate efficacy of 6-OHDA lesions and grafted dopaminergic neurons response to amphetamine, post synaptic dopaminergic receptor sensitivity using apomorphine, activation of grafted dopaminergic neurons expressing DREADDs using CNO and potency of 5HT agonists (5HT-4, 5HT-6, 5HT-7) acting on grafted tissue. Rats were placed in automated rotometer bowls modelled after the design of (Ungerstedt and Arbuthnott, 1970) and recorded using a rotation software (AccuScan Instruments Inc.). For all rotations rats were allowed to habituate in the rotometer bowls for 10 minutes prior to injection. Amphetamine acts as a DAT inhibitor and facilitates DA release, and induces rotational behavior in unilateral 6-OHDA lesioned animals that correlates well to cell loss in the SN. Following habituation, the animals were recorded for a total of 90 minutes following amphetamine injection (2.5 mg/kg i.p.). Apomorphine, which acts as a potent postsynaptic DA agonist and evaluates DA receptor sensitivity in unilateral 6-OHDA animal models, was used in order to evaluate if grafted dopaminergic neurons normalize receptor supersensitivity. Following habituation, animals were injected with 0.05 mg/kg apomorphine (s.c.) and recorded for a total of 40 minutes. In order to assess the rotational behavior response to activation of dopaminergic neurons expressing the hM-3Dq + rM3Ds DREADD receptors, CNO was injected (3 or 10 mg/kg s.c.) following habituation and recorded for a total of two hours.

Stepping test

As a potent nigral 6-OHDA lesion produces forelimb akinesia in animal models, we investigated if the grafted dopaminergic neurons could be modulated in a bi-directional fashion using the hM3Dq + rM3Ds neuronal activating and KORD silencing DREADDs in the stepping task initially described by (Schallert *et al.,* 1979), modified to a side-stepping test by (Olsson *et al.,* 1995). Animals were trained to be held by the researcher whilst making adjusting forelimb steps as the animal was moved sideways across a flat surface at a constant speed for a total length of 90 cm. The number of adjusting steps was then counted for both forehand and backhand movements and compared intact forelimb (right) to the lesioned forelimb (left). Once a stable

baseline was achieved the animals were injected with CNO (10mg/kg (s.c.), SalB (10mg/kg s.c.) or a vehicle and tested at 60 or 15 minutes post injection respectively for 3 consecutive trials. The researcher was blinded to both animal group and treatment.

Cylinder task

Unilateral 6-OHDA lesioned animals exhibit a strong exploratory forelimb asymmetry as previously described (Bjorklund *et al.,* 2010). In order to determine the potency of the 6-OHDA lesions as well as investigate if modulation of dopaminergic grafts may influence asymmetric forelimb behavior, animals were placed in a glass cylinder (20 cm in diameter) and recorded with a digital video camera under low light conditions. Two perpendicular mirrors were placed behind the cylinder, allowing for a clear visualization of the animals from all angles within the cylinder. The animals were recorded for at least 30 touches between paw and the walls of glass cylinder, or for a maximum of 5 minutes following injection of CNO (10mg/kg), SalB (10mg/kg) or a vehicle. Animals were tested at 60 or 15 minutes post injection for CNO or SalB, respectively. A blinded researcher scored the animals post hoc, with the score expressed as percentage of ipsilateral (right) or contralateral (left) touches out of the number of total touches.

Assessment of sensorimotor function

Rats develop a lateralized sensorimotor neglect following a 6-OHDA lesion. To investigate the result of the 6-OHDA lesion and the effect of modulation of dopaminergic neurons within the graft using DREADDs, rats were tested in the three different tests of sensorimotor function: Corridor test, Disengage behavior and Sensorimotor orientation.

Corridor task | The rats were placed inside a corridor (1500 x 70 x 230 mm) with ten pairs of adjacent food bowls evenly distributed within the corridor as described previously (Dowd *et al.,* 2005). Each food bowl was filled with 5-10 sugar pellets. Retrievals from the food bowl were defined as each time the rats poked their nose into a unique bowl. Repeated nose pokes into the same bowl without any retrieval from other bowls was not scored. The rats were tested until 20 retrievals were scored, or for a maximum of five minutes. Prior to testing the rats were trained in the corridor for five days until a stable baseline was achieved. Before each test, rats were habituated in an empty corridor without any food bowls for five minutes. A blinded researcher scored rats by percentage ipsilateral (right) and contralateral (left) retrievals out of the total number of retrievals. Animals were tested at 60 minutes post injection of CNO (10mg/kg) and 15 minutes post injection of SalB.

Disengage task | Animals were placed on an elevated platform and given pieces of milk chocolate as descried previously (Mandel *et al.,* 1990; Winkler *et al.,* 2000). Animals were trained for two consecutive days in order to start eating the chocolate. Once the animals were eating a piece of chocolate, the perioral region beneath the vibrissae on each side of the head was repeatedly touched with the use of a wooden probe. This was done until the animal disengaged from eating the chocolate and respond to the wooden probe and the latency recorded to the nearest second. Immediate response to the probe was scored as one second while no response was scored for a maximum of 180 seconds. Animals were scored 60 minutes post CNO injection (10mg/kg S.C.).

Sensorimotor orientation | Animals were placed on an elevated platform and assessed for two consecutive days based on a modified protocol from (Mandel *et al.,* 1990). Each rat was tested by a 'blind' experimenter who used a pointed, but blunt, wooden probe such that the rat could neither see nor hear the approach of the probe prior to it contacting the perioral region (basically the area of skin beneath the vibrissae). The animal was then touched with the probe on one side of the face or the other and repeatedly stimulated at 1-2 s intervals. The latency to respond to the probe was recorded to the nearest second (an immediate response was recorded as 1 s). An orienting response was recorded if the rat turned toward the stimulus and contacted the probe. Perioral stimulation in a particular sensory hemifield was discontinued if a rat did not respond within 30 s (then recorded as 30 seconds). Animals were scored 60 minutes post CNO injection (10mg/kg s.c.) or 15 minutes post SalB injection (10mg/kg s.c.).

Drug preparation

6-OHDA (Cl-salt, Sigma-Aldrich) was prepared in sterile 0.9 % saline with the addition of 0.02 % ascorbic acid to prevent oxidization and vortexed until fully dissolved. d-Amphetamine for all behavioral tests was prepared at 1 mg/ml in sterile 0.9 % saline solution and vortexed until fully dissolved. d-Amphetamine was then injected i.p. at 2.5 mg/kg. Apomorphine (Apoteksbolaget) was prepared at 1mg/ml solution in sterile 0.9 % saline with the addition of 0,02 % ascorbic acid in order to prevent rapid oxidation and vortexed until fully dissolved. Apomorphine was injected into animals S.C. at 0.05 mg/kg. CNO (Toronto Research Chemicals) was pre-diluted in pure DMSO (Sigma) (ml = 2% of final volume) and vortexed until fully dissolved into a clear yellow solution. This was then diluted in sterile 0.9 % saline to 3 mg/ml with 2 % DMSO and injected s.c. at 3 or 10 mg/kg. SalB (Cayman Chemical Company) was prepared in pure DMSO at 30 mg/ml and vortexed extensively until fully dissolved. Animals were then injected s.c. at 10mg/kg.

Abnormal involuntary movements (AIMS)

We found that treating grafted animals with CNO or the 5-HT6 agonist ST-1936 induced rotational behavior. In order to determine if this emulated graft-induced dyskinesia, animals were assessed for abnormal involuntary movements as described previously (Cenci and Lundblad, 2007). Animals were put into empty transparent cages and were habituated for a total of 10 minutes prior to the start of the test. After an initial baseline AIMs score, animals were injected with CNO (3mg/kg s.c.) or ST-1936 (20 mg/kg i.p.). The animals were scored for a total of two hours post injection, with each animal being scored every 20 minutes for 1 minute, resulting in each animal being scored for AIMs a total of seven time points. Animals were scored for limb, axial and orolingual AIMs as well as general locomotion, using the rating scale developed by Cenci & Lundblad (Cenci and Lundblad, 2007).

Laser scanning confocal microscopy

In order to assess co-localization analysis, laser scanning confocal microscopy was conducted using a Leica SP8 microscope. All confocal images were captured using a HyD detector and always with the lasers activated in sequential mode in order to avoid fluorescence signal bleed through. Solid-state lasers at wavelengths of 405, 488, 552 and 650 nm were utilized to excite the respective fluorophores. The pinhole was retained at Airy 1 for all image acquisitions. Post acquisition, deconvolution was performed using the "Deconvolution" plugin for ImageJ (developed by the Biomedical Imaging Group [BIG] - EPFL – Switzerland http://bigwww.epfl.ch/) utilizing the Richardson-Lucy algorithm and applying point-spreads functions (PSFs) calculated for the specific imaging equipment using the Gibson and Lanni model in the PSF Generator (BIG, EPFL – Switzerland http://bigwww.epfl.ch/algorithms/psfgenerator/).

Stereological quantification

The number of TH- and mCherry-, HA- and GFP-positive cells in the striatum as well as TH-positive neurons in the Substantia nigra was quantified using the Stereo Investigator software suite (version 11) from MBF bioscience with a 100 x magnification oil lens (numerical aperture: 1.40). The total numbers of cells were estimated according to the optical fractionator (West 1999) and the coefficient of error was calculated according to Gundersen *et al.,* (Gundersen & Jensen 1987), and values ≤ 0.05 were accepted.

Statistics

Data analysis, plotting and statistics were conducted in R Statistical Computing Platform (version 3.2.1) and SPSS version 23. Statistical tests included Bonferroni corrected paired Student's t-test when only two states/time points were compared and one-way ANOVA or two-way mixed model repeated measures ANOVA when three or more groups/states/time points were compared. In the latter case this is followed by Levene's Homogeneity test. This determined the choice of post-hoc test to either Dunnett's T3 (when Levene's fail) or Tukey's LSD. For assays where the results displayed a skewed, non-normal, distribution, the ANOVA was replaced by the Kruskal-Wallis test followed by Bonferroni corrected all pair comparison using the Mann-Whitney U test. Unless otherwise noted, data in figures is presented as the arithmetic mean plus and/or minus one standard error of the mean (SEM). Comparisons were considered significant when the multiple comparison corrected p-value was less than 0.05.

Electrochemical Detection

High-speed chronoamperometric measurements (5 Hz) of extracellular DA levels were performed using a Pentium-IV microcomputer-controlled instrument (FAST-16; Quanteon) see (Hoffman and Gerhardt, 1998)). Briefly, a square wave potential was applied (+0.55 V; resting 0.0 V vs. Ag/AgCl reference), and oxidation/reduction currents were recorded with Nafion (Sigma Aldrich) coated, single carbon fiber electrodes (diameter approx. 20-30 µm; length approx. 100-150 µm) (Quanteon, KY, US). Before *in vivo* recordings each electrode was calibrated in 0.1M PBS (pH=7.4) to determine the electrodes sensitivity and linear response rate. Only electrodes that displayed a liner response rate to three 2µM increases of DA ($r^2 < .995$) were deemed linear. Furthermore, the electrodes selectivity ratio or DA to ascorbic acid was greater than 1000:1 with a limit of detection not higher than 0.01. After calibration the electrodes were mounted with the glass micropipettes used for local application of the respective compounds (KCL, CNO, SalB) using stick wax. The glass micropipettes with an outer diameter of 10-15mm were pulled in-house and fire polished. The distance to the electrode was 100 µm in average. For eliciting DA releases the respective micropipette was filled with either KCL (120mM, pH=7.4), CNO (100 µM), SalB (100 µM) and connected to a picospritzer (Aldax) micropressure system. An Ag/AgCl reference electrode was used which was prepared by electroplating an Ag wire in 1M HCl solution saturated with NaCl for at least 30 min before recording.

Electrode Implantation and Recording Procedures

Recordings were from the center of the three transplants were done after all behavioral data had been collated. We here performed the electrochemical recordings under gaseous isoflurane anesthetic with the animals' skull being secured in a stereotaxic frame. The animals' body temperature was kept constant at 37°C using a heating pad during the entire procedure. With the reference electrode being placed under the skin, the recording electrode/micropipette assembly was lowered into the brain at the following coordinate: AP/ML/DV. After insertion into the brain the electrodes were allowed to calibrate for at least 30 min before pressure injecting 4 applications of KCL (250nL, 10-40psi, 0.1-0.6s). Initial interval trials of 3, 5, 7 and 10 minutes have shown that a 7-minute interval was sufficient for the system to recover and to generate a full comparable DA release between 4 applications. Subsequently each recording was based on at least 3 applications of KCL with 7-minute intervals followed by the test compound (CNO/SalB) after a 7-minute interval. To test the neuronal silencing of the Gi receptor we pressure injected SalB at the relative peak DA release for CNO (see Fig. 3B) as well as 60 seconds before local application of KCL (Fig. S6D) and CNO (Fig. S6F). Recordings lasted typically between 6 and 10 hours, depending on compound used and responses generated.

SUPPLEMENTAL REFERENCES

Bjorklund, T., Carlsson, T., Cederfjall, E.A., Carta, M., and Kirik, D. (2010). Optimized adeno-associated viral vector-mediated striatal DOPA delivery restores sensorimotor function and prevents dyskinesias in a model of advanced Parkinson's disease. Brain 133, 496-511.

Brown, A.J., Fisher, D.A., Kouranova, E., McCoy, A., Forbes, K., Wu, Y., Henry, R., Ji, D., Chambers, A., Warren, J., *et al.* (2013). Whole-rat conditional gene knockout via genome editing. Nat Methods 10, 638-640.

Cenci, M.A., and Lundblad, M. (2007). Ratings of L-DOPA-induced dyskinesia in the unilateral 6-OHDA lesion model of Parkinson's disease in rats and mice. Curr Protoc Neurosci Chapter 9, Unit 9 25.

Dowd, E., Monville, C., Torres, E.M., and Dunnett, S.B. (2005). The Corridor Task: a simple test of lateralised response selection sensitive to unilateral dopamine deafferentation and graft-derived dopamine replacement in the striatum. Brain Res Bull 68, 24-30.

Grealish, S., Diguet, E., Kirkeby, A., Mattsson, B., Heuer, A., Bramoulle, Y., Van Camp, N., Perrier, A.L., Hantraye, P., Bjorklund, A., and Parmar, M. (2014). Human ESC-derived dopamine neurons show similar preclinical efficacy and potency to fetal neurons when grafted in a rat model of Parkinson's disease. Cell Stem Cell 15, 653-665.

Gundersen, H.J., and Jensen, E.B. (1987). The efficiency of systematic sampling in stereology and its prediction. J Microsc 147, 229-263.

Hagell, P., Piccini, P., Bjorklund, A., Brundin, P., Rehncrona, S., Widner, H., Crabb, L., Pavese, N., Oertel, W.H., Quinn, N., *et al.* (2002). Dyskinesias following neural transplantation in Parkinson's disease. Nat Neurosci 5, 627-628.

Hoffman, A.F., and Gerhardt, G.A. (1998). In vivo electrochemical studies of dopamine clearance in the rat substantia nigra: effects of locally applied uptake inhibitors and unilateral 6-hydroxydopamine lesions. J Neurochem 70, 179-189.

Kirkeby, A., Nelander, J., and Parmar, M. (2012). Generating regionalized neuronal cells from pluripotency, a step-by-step protocol. Front Cell Neurosci 6, 64.

Lindvall, O., Widner, H., Rehncrona, S., Brundin, P., Odin, P., Gustavii, B., Frackowiak, R., Leenders, K.L., Sawle, G., Rothwell, J.C., and et al. (1992). Transplantation of fetal dopamine neurons in Parkinson's disease: one-year clinical and neurophysiological observations in two patients with putaminal implants. Ann Neurol 31, 155-165.

Mandel, R.J., Brundin, P., and Bjorklund, A. (1990). The Importance of Graft Placement and Task Complexity for Transplant-Induced Recovery of Simple and Complex Sensorimotor Deficits in Dopamine Denervated Rats. Eur J Neurosci 2, 888-894.

Olsson, M., Nikkhah, G., Bentlage, C., and Bjorklund, A. (1995). Forelimb akinesia in the rat Parkinson model: differential effects of dopamine agonists and nigral transplants as assessed by a new stepping test. J Neurosci 15, 3863-3875.

Piccini, P., Brooks, D.J., Bjorklund, A., Gunn, R.N., Grasby, P.M., Rimoldi, O., Brundin, P., Hagell, P., Rehncrona, S., Widner, H., and Lindvall, O. (1999). Dopamine release from nigral transplants visualized *in vivo* in a Parkinson's patient. Nat Neurosci 2, 1137-1140.

Schallert, T., De Ryck, M., Whishaw, I.Q., Ramirez, V.D., and Teitelbaum, P. (1979). Excessive bracing reactions and their control by atropine and L-DOPA in an animal analog of Parkinsonism. Exp Neurol 64, 33-43.

Ungerstedt, U., and Arbuthnott, G.W. (1970). Quantitative recording of rotational behavior in rats after 6-hydroxy-dopamine lesions of the nigrostriatal dopamine system. Brain Res 24, 485-493.

West, M.J. (1999). Stereological methods for estimating the total number of neurons and synapses: issues of precision and bias. Trends Neurosci 22, 51-61.

Winkler, C., Kirik, D., Bjorklund, A., and Dunnett, S.B. (2000). Transplantation in the rat model of Parkinson's disease: ectopic versus homotopic graft placement. Prog Brain Res 127, 233-265.

Zolotukhin, S., Potter, M., Zolotukhin, I., Sakai, Y., Loiler, S., Fraites, T.J., Jr., Chiodo, V.A., Phillipsberg, T., Muzyczka, N., Hauswirth, W.W., *et al.* (2002). Production and purification of serotype 1, 2, and 5 recombinant adeno-associated viral vectors. Methods 28, 158-167.
