# Supplementary figures and images for: DREADD Modulation of Transplanted DA Neurons Reveals a Novel Parkinsonian Dyskinesia Mechanism Mediated by the Serotonin 5-HT6 Receptor
Source: Neuron. 2016 Jun 1;90(5):955–68. doi: 10.1016/j.neuron.2016.04.017 (PMC4893163; doi:10.1016/j.neuron.2016.04.017)

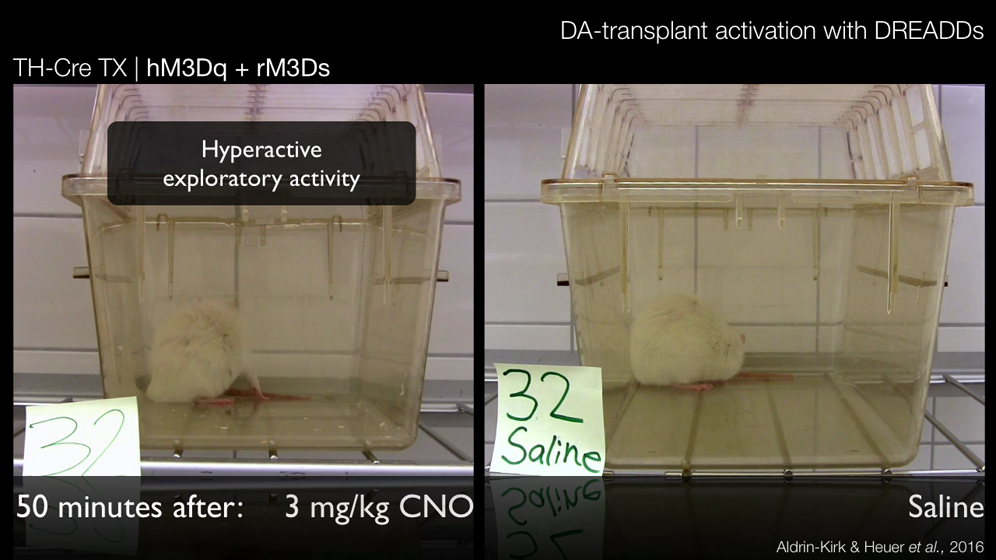

Supplement: Movie S1. Representative Video Recording of Abnormal Involuntary Movements Induced through Selective Increase of cAMP in the Fetal DA Transplant — Relates to Figure 4. The movie shows one representative animal from the hM3Dq + rM3Ds DREADD group. The movies are recorded 24 weeks following fetal grafting (15 weeks post-AAV injection). On 2 consecutive days, the animal was recorded in the empty cage after 3 mg/kg CNO (first day, left) or saline (second day, right). The two movies are time synced to display the same time point postinjection. Time points selected are representative for the developments of the different abnormal involuntary movements observed. The lesion-control animals develop only a transient locomotor increase, and the TH-TX hM3Dq + KORD animals displayed no change in behavior after CNO administration. [file mmc3.jpg]
